# Transcription factor 4 promotes increased corneal endothelial cellular migration by altering microtubules in Fuchs endothelial corneal dystrophy

**DOI:** 10.1038/s41598-024-61170-8

**Published:** 2024-05-04

**Authors:** Judy Yan, Shanti Mehta, Keya Patel, Narisa Dhupar, Ness Little, Stephan Ong Tone

**Affiliations:** 1 Sunnybrook Health Sciences Center and Sunnybrook Research Institute, 2075 Bayview Avenue, M-wing, 1st Floor, Toronto, ON M4N 3M5 Canada; 2 Department of Laboratory Medicine and Pathobiology, University of Toronto, Toronto, Canada; 3 Department of Ophthalmology and Vision Sciences, University of Toronto, Toronto, Canada

**Keywords:** Cell migration, Corneal diseases

## Abstract

Fuchs endothelial corneal dystrophy (FECD) is a complex corneal disease characterized by the progressive decline and morphological changes of corneal endothelial cells (CECs) that leads to corneal edema and vision loss. The most common mutation in FECD is an intronic CTG repeat expansion in transcription factor 4 (*TCF4*) that leads to its altered expression. Corneal endothelial wound healing occurs primarily through cell enlargement and migration, and FECD CECs have been shown to display increased migration speeds. In this study, we aim to determine whether TCF4 can promote cellular migration in FECD CECs. We generated stable CEC lines derived from FECD patients that overexpressed different TCF4 isoforms and investigated epithelial-to-mesenchymal (EMT) expression, morphological analysis and cellular migration speeds. We found that full length TCF4-B isoform overexpression promotes cellular migration in FECD CECs in an EMT-independent manner. RNA-sequencing identified several pathways including the negative regulation of microtubules, with *TUBB4A* (tubulin beta 4A class IVa) as the top upregulated gene. TUBB4A expression was increased in FECD ex vivo specimens, and there was altered expression of cytoskeleton proteins, tubulin and actin, compared to normal healthy donor ex vivo specimens. Additionally, there was increased acetylation and detyrosination of microtubules in FECD supporting that microtubule stability is altered in FECD and could promote cellular migration. Future studies could be aimed at investigating if targeting the cytoskeleton and microtubules would have therapeutic potential for FECD by promoting cellular migration and regeneration.

## Introduction

Fuchs endothelial corneal dystrophy (FECD) is a progressive, age-related, genetically complex disease with a female predilection that is the leading cause of corneal dysfunction. FECD affects 4–20% of the population over 40 years old and is the top indication for corneal transplantation worldwide^1,2^. FECD is characterized by corneal guttae, abnormal Descemet’s membrane (DM) thickening, and progressive corneal endothelial cell (CEC) loss. Mature CECs are post-mitotic and have a limited capacity to proliferate^3^. Instead, corneal endothelial wound healing occurs primarily through cell enlargement and migration^3–7^. While previous studies have investigated CEC migration behavior in normal corneal endothelium (CE)^8–11^ and immortalized cell lines^12–16^; we have previously explored CEC migration directly in FECD ex vivo patient specimens. We observed increased CEC migration speeds in FECD ex vivo specimens compared to healthy donor controls and noted that FECD CECs displayed more individual migration rather than collective sheet-like migration suggesting there is dysregulation of cell migration in FECD^17^. The significance of enhanced migratory speed and individual migration behavior of FECD CECs in disease pathobiology is currently unknown and requires further investigation. Particularly, since new approaches in treating FECD, such as Descemet’s stripping only (DSO) are based on CEC migration^18–21^.

FECD is a complex and heterogenous genetic disease, where a trinucleotide CTG repeat expansion within the third intron of transcription factor 4 (*TCF4)* is found in approximately 75% of FECD patients^1,22–24^. Proposed mechanisms for *TCF4-*CTG repeat expansion toxicity include *TCF4* dysregulation, gain-of-function with RNA foci/mis-splicing, repeat-associated non-AUG translation, and somatic instability^23,25–28^. Several studies have investigated the expression profile of TCF4 in FECD and have obtained differing results^27–33^. *TCF4-*CTG repeat expansion also impacts transcription initiated from nearby 5′ exons, leading to alternative TCF4 protein isoforms, with various levels of transcripts made from alternative promoters^31^. At least 18 different N-terminal sequences of TCF4 isoforms (A-R) have been identified, where full-length TCF4-B is best characterized and the roles of others remain to be elucidated^23,31,34^. A recent study has demonstrated that the CTG repeat expansion in TCF4 differentially modulates the activity of *TCF4* promoters, where nearby downstream *TCF4* promoter activity is decreased but expression of various other TCF4 transcripts are increased, possibly due to a compensatory mechanism^31^. Based on *TCF4* transcripts encoding specific TCF4 protein isoforms, the expression of transcripts encoding isoform TCF4-A, TCF4-B, TCF4-D and TCF4-H were increased in FECD, while TCF4-C was decreased^31^. Furthermore, RNA sequencing analysis of two independent FECD datasets indicated that *TCF4* expression was increased when measuring the expression of internal exons present in all *TCF4* transcripts, consistent with previous studies^32^. *TCF4* encodes E2-2, a transcription factor that dimerizes to bind E-box sequences (CANNTG) in the regulatory regions of target genes^23^. Additional TCF4 protein domains of importance include trans-activation domains, nuclear localization and export signals^23,31^. TCF4 target genes control diverse processes, including proliferation, differentiation, cell migration, and epithelial-to-mesenchymal transition (EMT)^23,35,36^. EMT guides cell migration/invasion, including the response to a pathological stress, such as wound healing, cancer progression, tissue fibrosis, and scar formation^36^. It has been previously shown that TCF4 can regulate EMT and promote cell migration^35^. There is thus strong evidence that TCF4 dysregulation contributes to FECD pathogenesis^1,22–24,32,37–40^, and may regulate cell migration^17,35^, but the mechanisms remain to be elucidated.

In this study, we investigated the hypothesis that TCF4 increases cellular migration in FECD CECs. We generated novel CEC lines derived from either healthy donor controls or FECD patients, that overexpressed different TCF4 isoforms and tested if this was sufficient to increase EMT or promote cellular migration. To investigate changes in gene expression with TCF4 overexpression, we performed bulk RNA sequencing on FECD CECs after stimulating cellular migration with a scratch assay and identified cytoskeleton regulators as potential mediators of the promigratory phenotype seen in FECD. We found that both actin and microtubules were dysregulated and observed that there were increased levels of acetylation and detyrosination of microtubules in FECD ex vivo specimens, suggesting that perturbation of microtubule dynamics can alter cellular migration in FECD.

## Results

**Figure 1:**
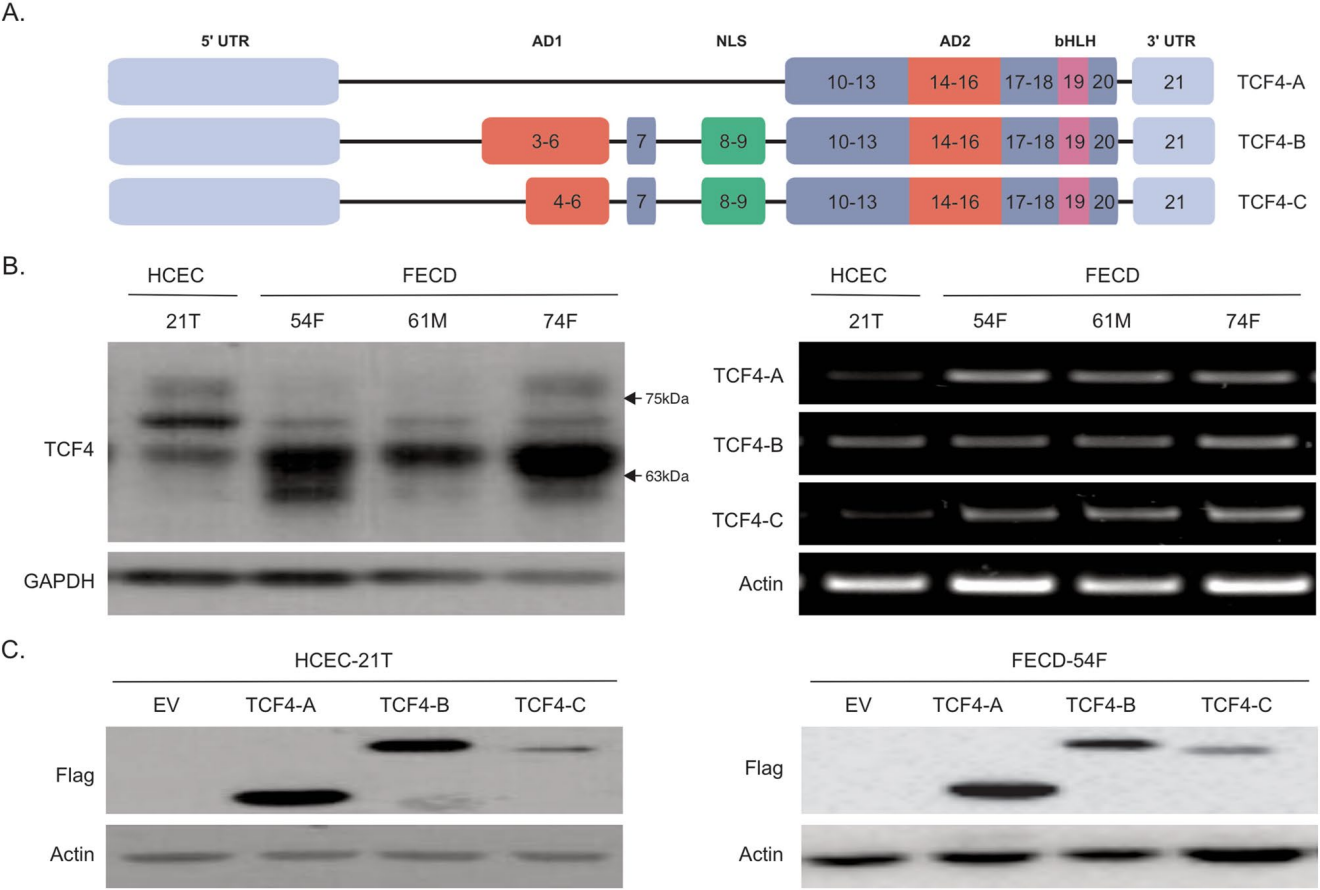
TCF4 expression in human corneal endothelial cells. (**A**) Illustration of TCF4 gene map for isoform -A, -B and -C. Schematic representation of the three isoforms with their corresponding exons (numbered) and domains (AD1-Activation domain 1 (red); NLS-Nuclear localization signal (green); AD2-Activation domain 2 (red); bHLH-basic helix-loop-helix (pink), UTR-untranslated region (light blue)) are shown. (**B**) Representative images of TCF4 protein (left) and mRNA (right) expression in a normal and FECD corneal endothelial cell lines. (**C**) Western blot analysis of flag expression in HCEC-21T and FECD-54F overexpressing exogenous flag-tagged TCF4 isoforms. Blots and gels have been cropped. Blots are converted to greyscale. Uncropped images are available in Supplemental Fig. S1.

TCF4 expression is the result of complex alternative splicing leading to a number of different isoforms varying in length. The TCF4 isoform that expresses all exons is expressed solely in testis while the more well studied TCF4-B isoform excludes exons 1 and 2^34^. Alternatively, shortened TCF4-A and TCF4-C isoforms lack exon 3 to 9 or only exon 3, respectively (Fig. 1A). We have previously reported that FECD CECs displayed increased cellular migration speeds, however TCF4 expression levels in these CECs were unknown^17^. We first investigated TCF4 expression by western blot analysis on CEC lines derived from normal healthy (HCEC-21T)^41^ or FECD patients (FECD-54F, FECD-61M, FECD-74F)^17^, and observed elevated TCF4 protein expression in FECD CECs (Fig. 1B, left panel, Supplemental Fig. S1A)). To detect the presence of specific TCF4 isoforms, we performed RT-PCR and detected TCF4-A, TCF4-B and TCF4-C using isoform specific primers (Fig. 1B, right panel, Supplemental Fig. S1B). To investigate if TCF4 isoform overexpression could promote EMT and cellular migration, we modified a normal healthy cell line (HCEC-21T) and a FECD cell line (FECD-54F) using lentivirus to stably overexpress different human flag-tagged TCF4 isoforms (-A, -B, -C) and GFP driven off separate CMV promoters (Fig. 1C, Supplemental Fig. S1C,D).

**Figure 2:**
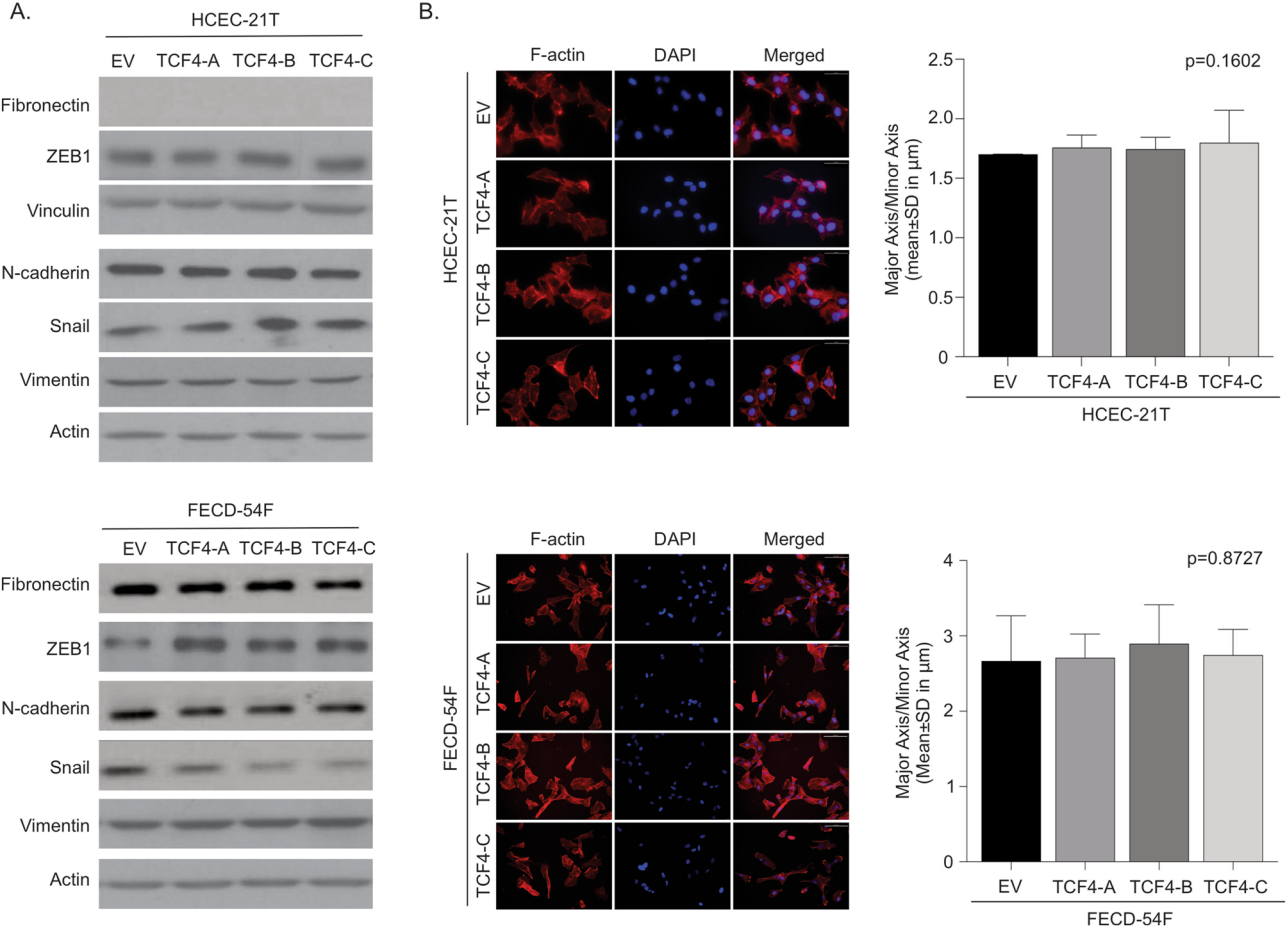
Overexpression of TCF4 does not affect EMT expression and morphology. (A) Western blot analysis of proteins involved in EMT in normal (top) and FECD (bottom) corneal endothelial cell lines overexpressing TCF4-A, -B or -C isoforms. Representative images are shown. β-actin was used as an internal control for FECD samples. β-actin and vinculin were used as internal controls for normal samples (B). Filamentous-actin (F-actin, red) and DAPI (blue) staining on normal and FECD corneal endothelial cell lines overexpressing TCF4-A, -B or -C isoforms. Representative images (left) and quantification (right, mean ± SD) of the major axis over the minor axis of cells are shown (scale bars; normal = 50 μm; FECD = 100 μm). SD, standard deviation. Blots have been cropped and converted to greyscale. Uncropped images are available in Supplemental Fig. S3 and Supplemental Fig. S4.

**Figure 3:**
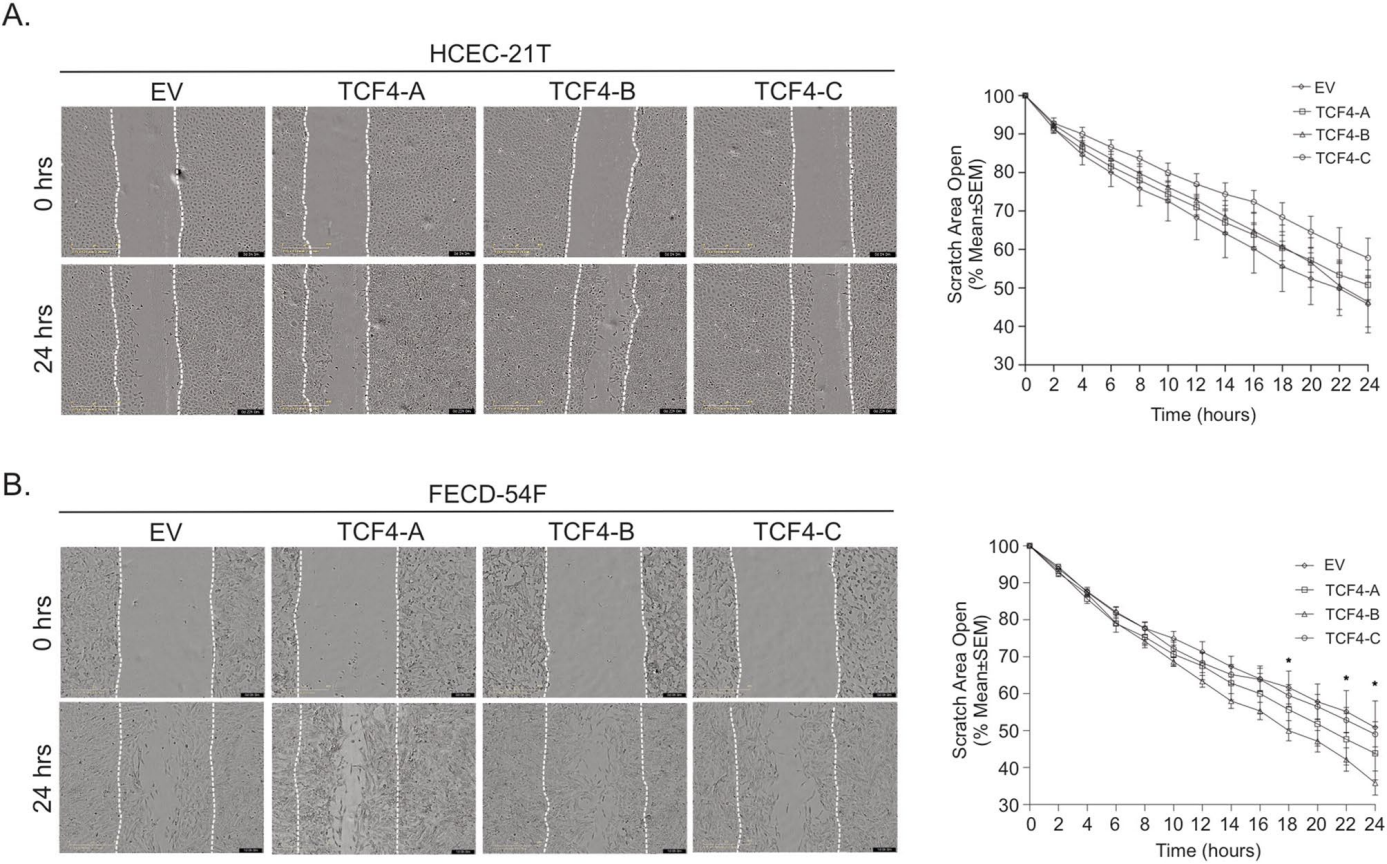
Overexpression of TCF4-B increases migration in FECD, but not normal CECs. Representative phase contrast images of HCEC-21T (A, left) and FECD-54F (B, left) for EV and TCF4 isoforms -A, -B, -C at 0 h and 24 h. Dashed line shows initial wound width at 0 h (scale bar = 400 μm). Quantification of wound closure measured by the percentage of the open scratch area for HCEC-21T (A, right) and FECD-54F (B, right) over 24 h (mean ± SEM). *p < 0.05 by 2-way ANOVA and dunnett’s post hoc test. EV = empty vector, SEM = standard error of the mean.

We performed western blot analysis probing for EMT markers (N-Cadherin, fibronectin, SNAI1, ZEB1, vimentin) and found that TCF4 isoform overexpression did not increase EMT marker expression in HCEC-21T and FECD-54F CECs (Fig. 2A, Supplemental Fig. S2, Supplemental Fig. S3, Supplemental Fig. S4). We next investigated if TCF4 isoform overexpression altered cellular morphology as detected by filamentous-actin (f-actin) staining and found no significant difference with TCF4 overexpression in HCEC-21T and FECD-54F CECs (Fig. 2B). To determine if TCF4 isoform overexpression altered cellular migration speeds, we performed a scratch assay and observed wound closure with live cell imaging over 24 h. We found that overexpression of TCF4 isoforms did not alter wound closure rates in HCEC-21T CECs (Fig. 3A). However, overexpression of full length TCF4-B isoform, but not TCF4-A or TCF4-C isoforms, in FECD-54F CECs resulted in increased cellular migration speeds compared to empty vector control at 24 h (35.8 ± 8.0% area open vs 50.8 ± 18.0% area open, p < 0.05) (Fig. 3B). Since overexpression of TCF4 isoforms did not increase EMT marker expression in FECD CECs, we investigated if TCF4-B increases cellular migration in an EMT-independent manner.

**Figure 4:**
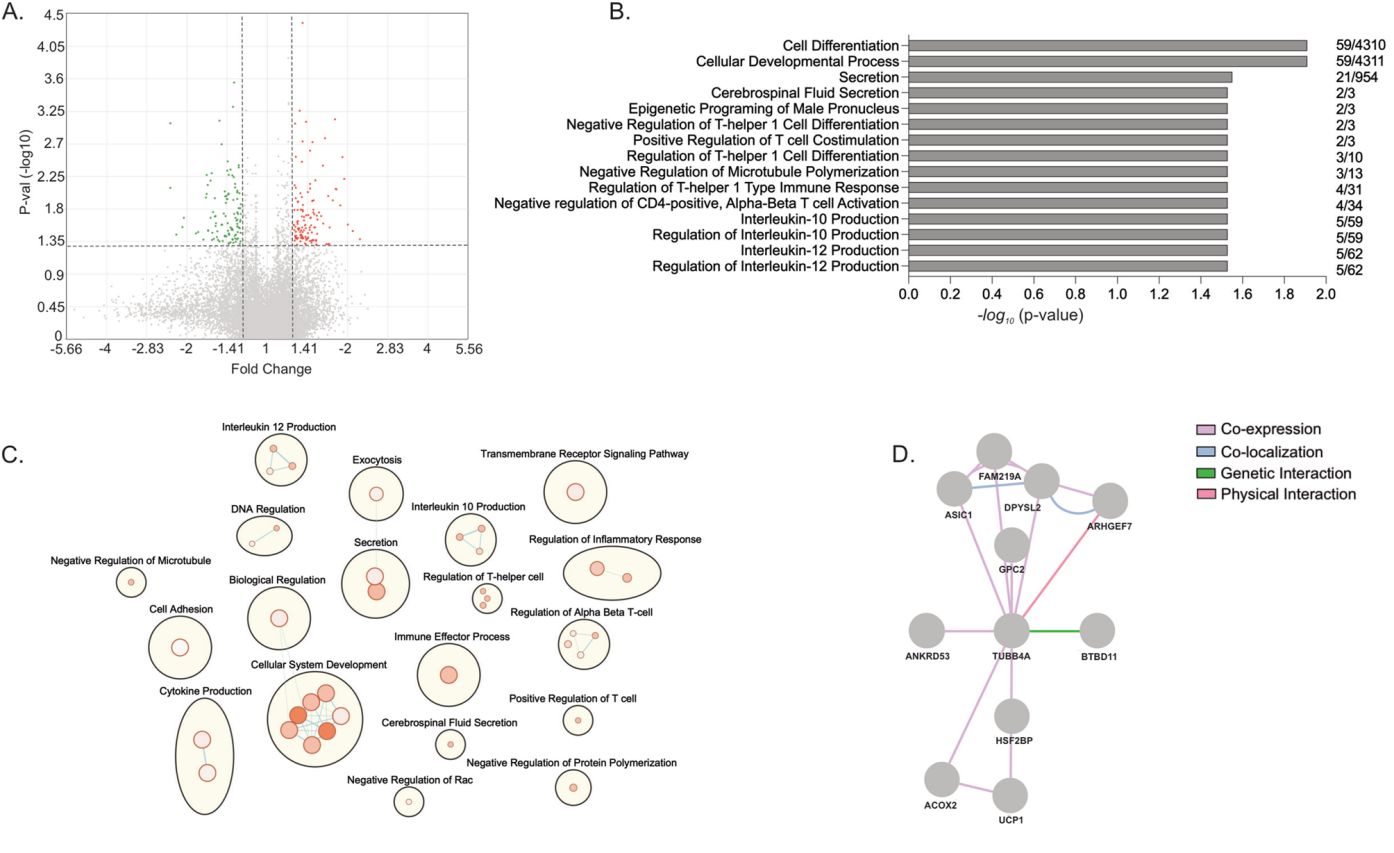
RNA sequencing reveal changes to gene expression following overexpression of TCF4-B in FECD-54F compared to empty vector control after stimulating cell migration with a scratch. (**A**) Volcano plot illustrating 121 upregulated (red) and 106 down regulated (green) genes at a *p* value cut off of less than 0.05 and fold change of 1.25 (dashed line). (**B**) Top 15 gene ontology (GO) annotations over-represented among the genes differentially expressed between FECD-54F over expressing TCF4-B compared to empty vector control. Number on the right of each GO term is the number of differentially expressed genes/total genes in the annotation. (**C**) Enrichment mapping of gene sets identified from the 227 differentially expressed genes. Nodes represent a gene set (GO term). Larger nodes correlates to the number of DEGs in a gene set. Shade of nodes correlates to a *p* value < 0.05. (Darker shade = smaller *p* value; lighter shade = *p* value closer to 0.05). Blue edges represent gene overlap between sets. Functionally related gene sets are clustered and circled together. (**D**) Gene–gene interaction predicted by GeneMANIA for TUBB4A and other DEGs. Edge color represents possible interaction between two genes.

**Figure 5:**
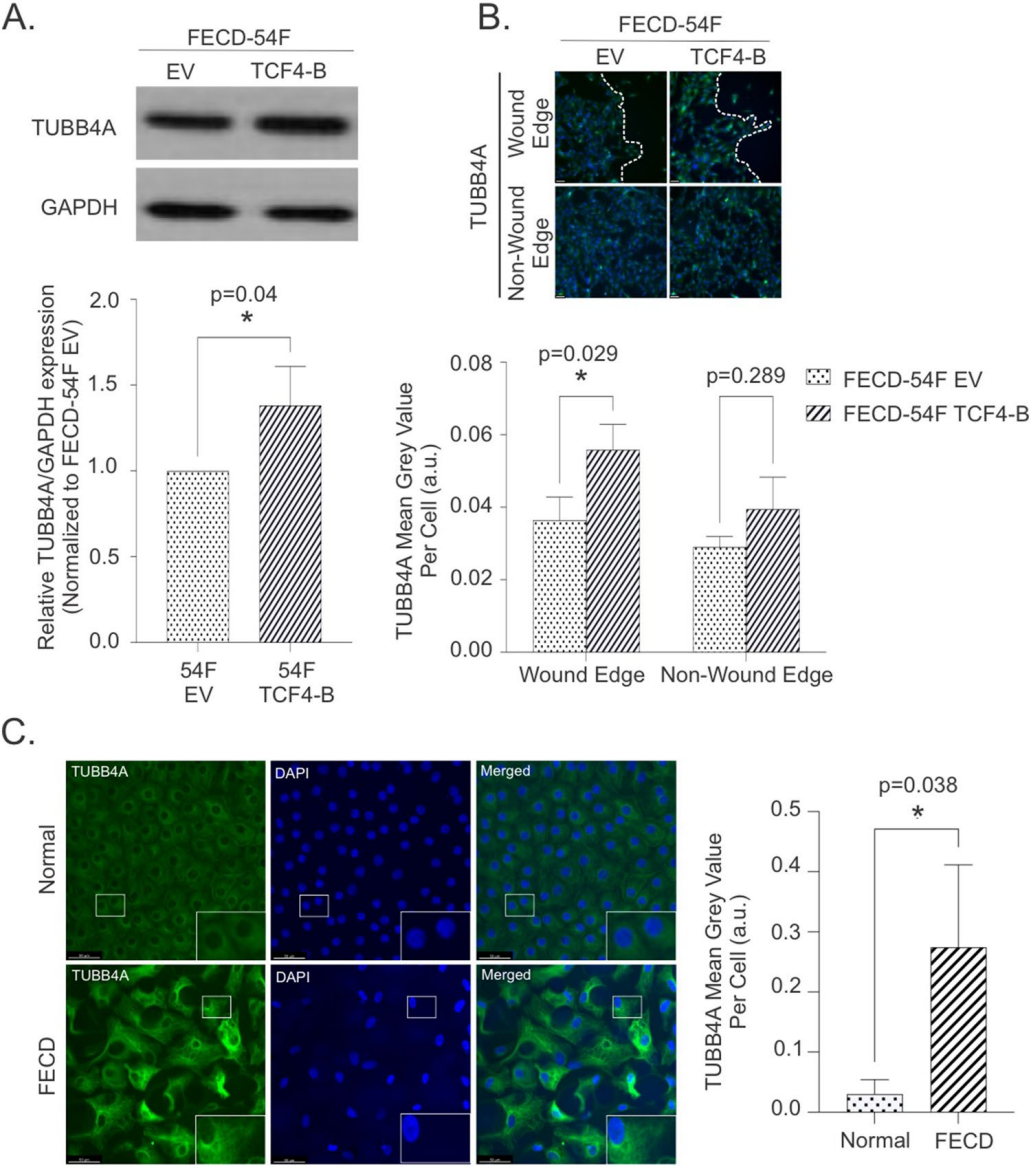
TUBB4A expression is increased in FECD-54F cells over expressing TCF4-B and in FECD ex vivo specimens. (**A**) Representative images (top) and densitometry quantification (bottom, mean ± SD) of TUBB4A protein expression by western blot in FECD-54F cells. (**B**) Representative immunofluorescence images (top, scale bar = 50 μm) and quantification (bottom, mean ± SD) of TUBB4A expression at the wound edge and non-wound edge between FECD-54F empty vector and overexpression of TCF4-B. (**C**) TUBB4A expression in normal (n = 3) and FECD ex vivo (n = 3) specimens. Representative images (left, scale bar = 50 μm) with 2.5 × magnification (inset) and fluorescence intensity quantification (right, mean ± SD) are shown. *p < 0.05 by two tailed student *t*-test. SD = standard deviation, EV = empty vector. Blots have been cropped and converted to greyscale. Uncropped images are available in Supplemental Fig. S6.

To identify differential expressed genes (DEGs) and potential pathways involved with mediating the increase in cellular migration speeds with TCF4-B overexpression in FECD-54F CECs, we performed bulk RNA sequencing (RNA-seq) after stimulating cell migration with a scratch (Fig. 4). We identified a total of 227 DEGs, with 121 upregulated and 106 downregulated DEGs with TCF4-B overexpression compared to control vector (Fig. 4A, Supplemental Fig. S5, Supplemental Table S1). Using g:Profiler we identified 38 GO annotations over-represented in our gene list (Fig. 4B, top 15 annotations displayed). To further examine pathways enriched in our gene list, we performed a pathway enrichment analysis using Cytoscape and identified an enrichment of the immune response, cellular and developmental regulation, and secretion amongst the top pathways (Fig. 4C). To further investigate if cytoskeleton regulators and potential mediators of cellular migration were involved, our GO analysis revealed that negative regulation of microtubules was one of the pathways identified (Fig. 4C). We identified 3 DEGs related to the negative regulation of microtubules, with *TUBB4A* (tubulin beta 4A class IVa), as the top upregulated DEG, followed by *ARHGEF7* and *MAP2*. To predict possible gene interactions, we performed an analysis using GeneMANIA and determined that TUBB4A interacts with several DEGs identified in our data set (Fig. 4D). To validate our RNA-seq data showing increased *TUBB4A* in FECD-54F CECs overexpressing TCF4-B after stimulating cell migration with a scratch, we performed western blot analysis and found increased TUBB4A protein expression in FECD-54F CECs (Fig. 5A, Supplemental Fig. S6) and increased TUBB4A intensity along the wound edge in FECD-54F CECs (Fig. 5B). We next validated these findings and observed increased TUBB4A intensity in FECD ex vivo specimens compared to normal healthy donor ex vivo specimens (Fig. 5C).

**Figure 6:**
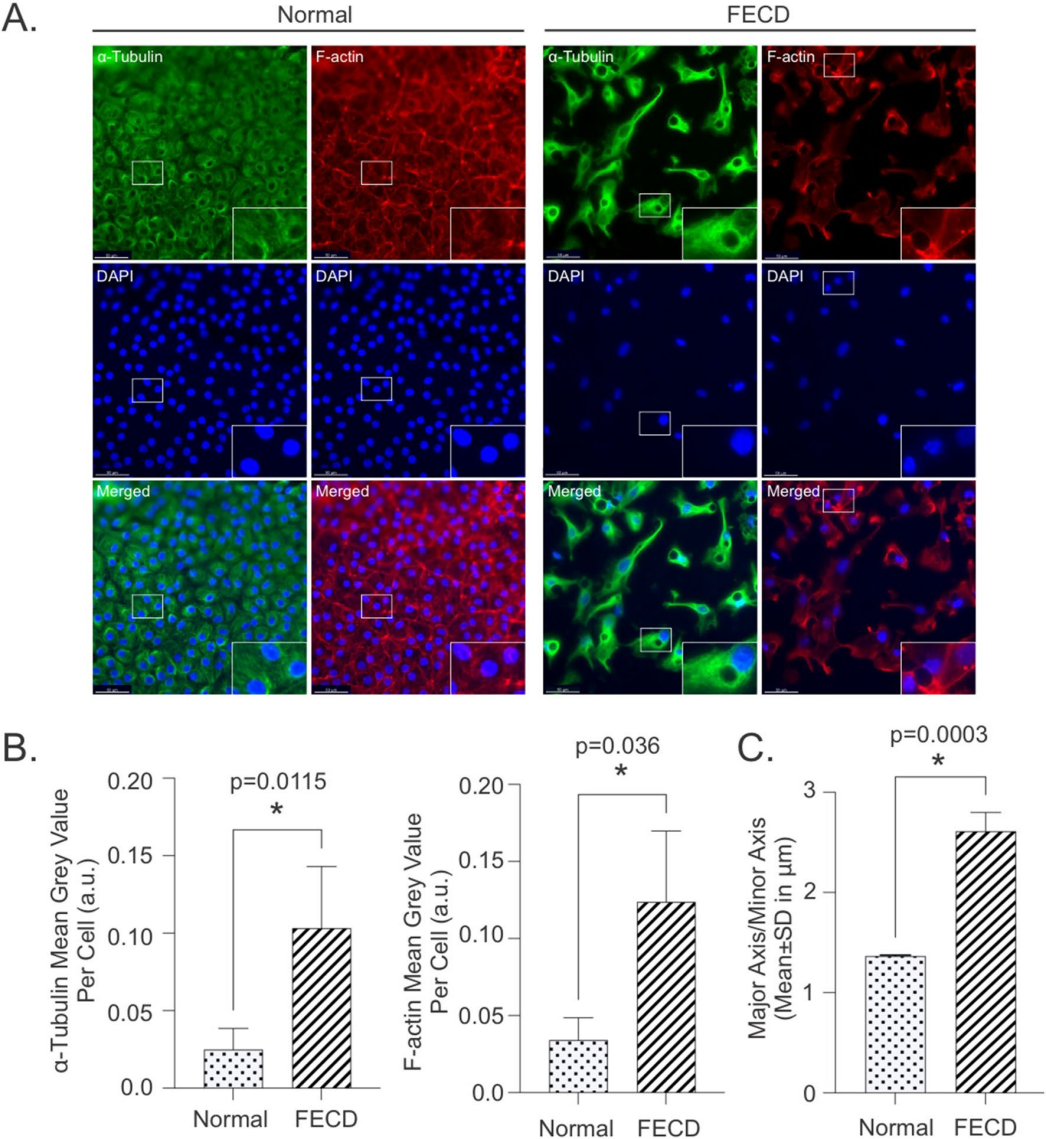
FECD ex vivo specimens display increased α-tubulin and filamentous actin (F-actin) expression along with more fibroblastic-like morphological features. (**A**) Representative images with 2.5 × magnification (inset) of α-tubulin and F-actin staining in normal (n = 3) and FECD (n = 3) ex vivo specimens (scale bar = 50 μm). (**B**) Fluorescence intensity quantification and (**C**) quantification of cell morphology with measurements of the major axis over the minor axis of cells are shown (mean ± SD) for FECD and normal. *p < 0.05 by two tailed student *t*-test. SD = standard deviation.

**Figure 7:**
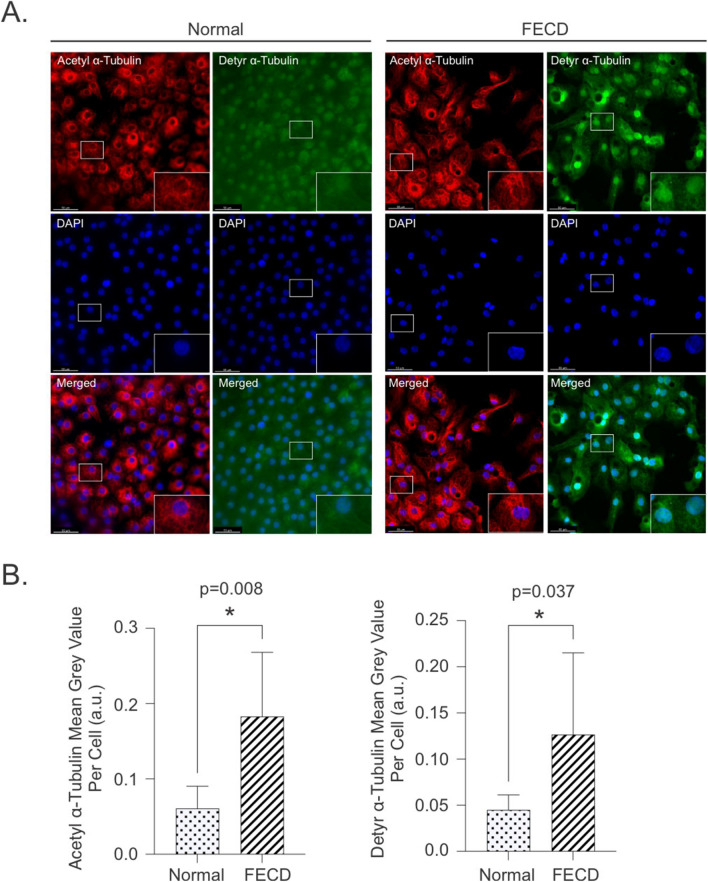
Increased expression of acetylated and detyrosinated α-tubulin in FECD ex vivo specimens. (**A**) Representative images with 2.5 × magnification (inset) of acetylated and detyrosinated α-tubulin staining (scale bar = 50 μm) and (**B**) quantification of fluorescence intensity (mean ± SD) are shown for FECD (n = 3) and normal (n = 4) ex vivo specimens. *p < 0.05 by two tailed student *t*-test. SD = standard deviation.

As *TUBB4A* encodes beta tubulin^42^ and is a cytoskeleton protein, we next investigated if the cytoskeleton is dysregulated in FECD. We immunostained FECD and normal healthy donor ex vivo specimens for α-tubulin and f-actin and observed that CECs from FECD ex vivo specimens displayed significant increases in α-tubulin and f-actin intensities compared to normal healthy donors (Fig. 6A,B). We also determined that CECs from FECD ex vivo specimens displayed altered cellular morphology when compared to normal controls (Fig. 6A,C). As the regulation of microtubules dynamics through post-translational modifications is important for cellular organization and function^43^, we investigated if acetylation and detyrosination of microtubules were increased in FECD. We found that there was increased acetylation and detyrosination of microtubules in FECD ex vivo specimens compared to normal healthy donor controls (Fig. 7A,B). Tubacin and parthenolide (PTL) have been reported to modulate acetylation and detyrosination of microtubules, respectively and was used as a control for our immunofluorescence staining. Tubacin has been reported to increase acetylated tubulin by selectively inhibiting HDAC6^44^, whereas parthenolide has been reported to inhibit tubulin carboxypeptidase (TCP) and subsequently lower detyrosinated tubulin^45^. Addition of Tubacin in normal ex vivo tissues resulted in increased expression of acetylated α-tubulin (Supplemental Fig. S7A) compared to DMSO. However, the addition of PTL in normal ex vivo tissues resulted in an observed lower cytoplasmic staining for detyrosinated α-tubulin but not statistically significant using our method of quantification (Supplemental Fig. S7B). Taken together, we provide supporting evidence that microtubule stability is altered in FECD and could promote cellular migration.

## Discussion

We have previously observed in ex vivo specimens that FECD CECs displayed increased migration speeds and individual cellular migration rather than collective migration patterns, suggesting an altered migratory response^17^. At first glance, increased CEC migration in FECD suggests that this response should be pro-regenerative, yet pathology develops^17^. In this study, we provide mechanistic data supporting that full length TCF4-B isoform can increase cellular migration speeds in FECD CECs, but not in healthy control CECs. This suggests that FECD CECs have an increased responsiveness to TCF4-B signaling compared to healthy control CECs, and that elevated TCF4-B levels in FECD may contribute to increased cellular migration speeds^31^.

TCF4 has been previously shown to be a regulator of EMT and cellular migration when overexpressed in MDCK epithelial cells^35^. EMT is a complex process that occurs during normal embryonic development to guide cell migration or invasion, or in response to a pathological stress, such as during wound healing, cancer progression, tissue fibrosis, and scar formation^36^. During EMT, there is a loss of intercellular contacts and cellular polarity, and acquisition of a fibroblastic pro-migratory phenotype. EMT generates mesenchymal-like cells that upregulate EMT genes and produce excessive collagen-rich extracellular matrix (ECM). There is increasing evidence that implicates abnormal ECM regulation and EMT in FECD pathogenesis^37–40,46–49^. In both FECD ex vivo specimens and in vitro, there is an upregulation of EMT markers (SNAI1, CDH2, ZEB1, FN1, TGFβI), and acquisition of an abnormal fibroblastic morphology with loss of organized junctional staining of CDH2 and hexagonal CEC mosaic^38–40^. While mechanistically, ectopic *TCF4* can drive EMT^35^, we found that that TCF4 overexpression was insufficient to promote further EMT in FECD CECs as detected by EMT protein markers and morphological analysis. However, we did observe that TCF4-B overexpression increased cellular migration speeds in an EMT-independent pathway in FECD CECs by regulating the cytoskeleton.

The cytoskeleton, which is primarily composed of actin microfilaments, microtubules, and intermediate filaments, plays an integral role in cellular migration and wound repair^50,51^. In the corneal endothelium, following circular freeze injury, a wound response is initiated with the presence of stress fibers and microtubule reorganization in CECs^52^. This injury-induced CEC migration response appears to be more dependent on microtubules than actin microfilament reorganization as exposure to colchicine, an inhibitor of microtubule polymerization, impedes wound healing, whereas cytochalasin B, an inhibitor of actin polymerization, slows down but does not prevent wound healing^52^. Microtubules are hollow tubular structures composed of heterodimers of alpha and beta-tubulin and are part of the cytoskeleton that plays an important role in cell migration, polarization, intracellular trafficking and cell division^43,50^. Cellular migration or locomotion is achieved through the polarization of microtubules at the leading edge of the cell in the direction of migration^50^. Our data also supports that the cytoskeleton is important in cellular migration as our RNA sequencing analysis identified regulators of microtubules as top DEGs after stimulating a wound healing response in FECD CECs overexpressing TCF4-B. We confirmed that both TUBB4A and the cytoskeleton (actin and tubulin) were dysregulated in FECD ex vivo specimens. There are numerous genes encoding for tubulin proteins, where the major tubulin subclasses are alpha and beta-tubulin, and TUBB4A is part of the beta tubulin family^42,53^. There are a large number of tubulin genes that are expressed during neurodevelopment which suggests that different tubulin isoforms may be required for specific microtubule functions during different stages such as neuronal migration^54^. TUBB4A is predominantly expressed in the brain and has been implicated with malformation of cortical development and dystonia^42,53,55^. TUBB4A is also highly expressed in human prostate cancer and melanoma cells, and *TUBB4A* knockout results in reduced cell growth and migration^56,57^. These studies support TUBB4A as an important regulator of cellular migration both during development and in metastatic invasion. Here, we demonstrate that TUBB4A expression is increased in FECD ex vivo specimens, and that TUBB4A expression is regulated through TCF4-B signalling. We also found that there was increased tubulin acetylation and detyrosination in FECD ex vivo specimens, which are important posttranslational modifications that are associated with stabilized microtubules that can accumulate in the protrusion of migrating cells^50,58^.The disruption of microtubule dynamics has also been associated with both increased oxidative stress and TNF-α signaling, leading to disassembly of microtubules and loss of barrier function in CECs^59,60^. Collectively, there is increasing evidence that regulators of microtubules are important for CEC function. Our RNA-seq dataset also identified *MAP2* (microtubule-associated protein 2) and *ARHGEF7* (Rho guanine nucleotide exchange factor 7) as top DEGs involved with the regulation of microtubules. MAP2 plays an important role in neuronal morphogenesis and mediating interactions between actin and microtubules and is associated with stable microtubules^61^. ARHGEF7, also known as Beta-PIX, can activate Rho proteins Rac1 and Cdc42 to regulate cellular migration of corneal epithelial cells during wound healing^62^. However, whether MAP2 and ARHGEF7 are implicated in FECD and the CEC wound healing response remains to be investigated.

One limitation of our study is that we used immortalized cell lines derived from FECD patients. We attempted to validate these findings directly in FECD primary cell cultures but were unable to consistently establish these cell cultures in sufficient quantities. The difficulty in establishing FECD primary cell cultures is well known, and has been attributed to a limited mitotic capacity and limited ability to passage them as they undergo rapid senescence or endothelial to mesenchymal transition^63^. However, we did confirm the cytoskeleton changes directly in FECD *ex-vivo* specimens, supporting our findings from FECD cell lines. These findings in combination with our previously published findings that FECD CECs displayed increased migration speeds in FECD *ex-vivo* specimens and FECD cell lines with CTG repeat expansion in TCF4^17^ support that the increase in cell migration in FECD CECs is not a cell line specific effect.

Corneal endothelial wound healing occurs primarily through cell enlargement and migration, rather than proliferation^3–11^. This regenerative wound healing response is stimulated with DSO, and functional recovery is based on CEC migration from the periphery to the central cornea^18–21^. Recently, two distinct migrating cell populations that display collective migration from the peripheral to central endothelium have been observed in outer corneal graft rims and a thermo-reversible hydrogel^9^. These two morphologically distinct populations were identified to be an early-onset migrating population triggered by disrupted contact inhibition and another late-onset population showing higher proliferative capacity and appearing less differentiated^9^. One possibility is that the peripheral corneal endothelium acts as a reserve to regenerate the corneal endothelium after injury or degeneration through these two populations, where the late-onset population may be derived from the progenitor rich transition zone^9,64–70^. Whether this occurs in vivo and if this migration pattern is altered in FECD remains to be investigated. As cellular migration in FECD is important, various pharmacological compounds such as ROCK inhibitors and an engineered analogue of FGF1, have been shown to promote CEC migration and regeneration *in vitro*^13,71^ and as adjuvant therapy for DSO^18^.

In summary, we report that TCF4-B promotes increased cellular migration in an EMT-independent manner by altering microtubules in FECD CECs, and that microtubule stability is dysregulated in FECD. Future studies could be aimed at investigating if targeting the cytoskeleton and microtubules could have therapeutic potential for FECD by promoting cellular migration and regeneration to restore corneal endothelium function.

## Materials and methods

### Cell lines

Immortalized FECD cell lines were generated as previously described^17^. In brief, CECs were isolated from a 54-year-old female (FECD-54F), a 61-year-old male (FECD-61M) and a 74-year-old female (FECD-74F) undergoing endothelial keratoplasty and SV-40 immortalized to generate cell lines. Cells were cultured in Chen’s Media consisting of Opti-MEM media (ThermoFisher Scientific, Waltham, MA) supplemented with 200 mg/L CaCl_2_ (Millipore Sigma, Oakville, Ontario), 0.08% chondroitin sulfate (Millipore Sigma, Oakville, Ontario), 50 µg/mL gentamicin (ThermoFisher Scientific, Waltham, MA), 1 × antibiotic/antimycotic (Wisent, St. Bruno, QC), 66 µg/mL bovine pituitary extract (Gemini, West Sacramento, CA), 5 ng/mL EGF (Millipore Sigma, Oakville, Ontario) and 8% fetal bovine serum (ThermoFisher Scientific, Waltham, MA). Immortalized normal HCEC-21T and FECD cell lines were graciously provided by Dr. Ula Jurkunas at The Schepens Eye Research Institute in Boston, Massachusetts^41^. Cell culture flasks or plates were coated with undiluted fibronectin coating mix (AthenaES, Baltimore, MD) prior to cell seeding and incubated at 37 °C and 5% CO_2_.

### Human tissues

The study was carried out according to the tenets of The Declaration of Helsinki and was approved by the Sunnybrook Health Sciences Centre Research Ethics Board (REB#5187 and REB#5070). Informed consent from patients undergoing surgery for FECD was obtained prior to tissue collection. Surgically removed tissues were stored in Optisol-GS (Bausch & Lomb) at 4 °C for 1–3 days. Normal tissues from donors were provided by The Eye Bank of Canada. See Supplemental Table S2 for patient and donor characteristics.

### Lentiviral overexpression of TCF4

Lentiviral vectors pLV[Exp]-EGFP:T2A:Hygro-CMV-TCF4/Flag expressing Human TCF4 isoforms A (NM_001243234.2), B (NM_001083962.2), and C (NM_001243227) were constructed by Vector Builder (Chicago, IL). pLV[Exp]-EGFP:T2A:Hygro-CMV-Flag vector without TCF4 was used as an empty vector control. Overexpression of TCF4 was carried out using a 2nd generation lentiviral packaging system, psPAX2 and pMD2.G. The packaging plasmids was transiently co-transfected with Lipofectamine 3000 (ThermoFisher Scientific, Waltham, MA) with the designed lentiviral plasmid into HEK293T. The virus containing media was harvested 48 h later, centrifuged to remove cell debris, and filtered through a 0.45 µM filter. Viral supernatant was added to cells for 24 h and selected for stable integration with hygromycin (200 µg/mL, Wisent, St. Bruno, QC).

### RNA transcriptomics

FECD-54F cells lines were seeded at a density of 150,000 cells per well in 12 well plates pre-coated with undiluted fibronectin coating mix and allowed to grow into a confluent monolayer for 48 h. A P-200 pipette tip was used to generate a linear scratch. Media was removed and replaced with fresh cell culture media. After 12 h, total RNA was extracted with PureLink RNA Mini Kit (ThermoFisher Scientific, Waltham, MA) according to the manufacturer’s instructions and sent to the Genomics Core Facility at the Sunnybrook Research Institute for bulk RNA sequencing. Samples were analyzed on a Bioanalyzer RNA Pico Assay and sequencing performed with the Ion Ampliseq Transcriptome Human Assay Sequencing. Briefly, cDNA library was constructed, and qPCR library quantified followed by sequencing template preparation on the Ion Chef Instrument. Sequencing was carried out with the Ion S5XL Next Generation Sequencing and data was analyzed using Transcriptome Analysis Console (TAC) Software to generate differentially expressed genes (DEGs) and heatmap clustering. Genes with fold change > 1.25 and < − 1.25 and *p* value < 0.05 were selected as DEGs. Functional analysis of differentially expressed genes was carried out using GeneMANIA^72^ to examine gene–gene interaction. Gene ontology and pathway enrichment analysis was carried out using g:Profiler^73^ and enrichment maps were generated with Cytoscape^74,75^.

### RT-PCR

Total RNA was extracted using TRIZOL (ThermoFisher Scientific, Waltham, MA) according to the manufacturer’s instructions. Reverse transcription was carried out using Superscript IV (ThermoFisher Scientific, Waltham, MA). Briefly, 2 µg of RNA was converted to cDNA at 65 °C for 5 min followed by a 1 min incubation on ice, 23 °C for 10 min, 50 °C for 10 min and 80 °C for 10 min. PCR primers and annealing temperatures are listed in Supplementary Table S3. The PCR reaction was carried out with DreamTaq Hot Start PCR Master Mix (ThermoFisher Scientific, Waltham, MA) at 95 °C for 3 min, 95 °C for 30 s, followed by 30 cycles at the annealing temperature for 30 s and 72 °C for 1 min.

### Scratch assay

HCEC-21T or FECD-54F cells lines were seeded at a density of 300,000 cells per well in 12 well plates pre-coated with undiluted fibronectin coating mix overnight. A P-200 pipette tip was used to generate a linear scratch. Media was removed and replaced with fresh cell culture media. Images were captured every 2 h at 10 × magnification for 24 h with the Incucyte S3 Live-Cell Analysis system (Sartorius). Scratch assay images were analyzed using ImageJ or T-Scratch.

### Western blot

HCEC-21T and FECD-54F CECs were plated in 100 mm cell culture plate pre-coated with undiluted fibronectin coating mix and allowed to grow for 48–72 h until cells reached 90–100% confluency at time of protein lysate collection. Whole cell lysates were prepared in a RIPA Buffer (50 mM Tris, pH 8, 150 mM NaCl, 5 mM EDTA, pH 8, 1% NP-40, 0.5% Sodium Deoxycholate, 0.1% SDS) containing 1 × protease inhibitor cocktail (Millipore Sigma, Oakville, Ontario) and 1 mM PMSF. Lysates were separated on an SDS-PAGE gel and transferred onto Amersham hybond ECL nitrocellulose membrane (Millipore Sigma, Oakville, Ontario). Membranes were blocked with 5% skim milk and subsequently incubated with the indicated antibodies at 4 °C overnight. Appropriate HRP-conjugated secondary antibodies were incubated for one hour at room temperature. Signals were detected using Pierce ECL Western Blotting Substrate (ThermoFisher Scientific, Waltham, MA) or Pierce SuperSignal West Pico Plus Chemiluminescent substrate (ThermoFisher Scientific, Waltham, MA). The primary and secondary antibodies and the concentrations used were: rabbit anti-TCF-4 (1:1000, Proteintech, Rosemont, IL), rabbit anti-Flag (1:1000, Cell Signaling, Danvers, MA), mouse anti-Fibronectin (1:1000, Santa Cruz, Dallas, TX), rabbit anti-ZEB1 (1:1000, Cell Signaling, Danvers, MA), mouse anti-N-cadherin (1:1000, Santa Cruz, Dallas, TX), rabbit anti-Snail (1:1000, Cell Signaling, Danvers, MA), mouse anti-Vimentin (1:1000, Santa Cruz, Dallas, TX), mouse anti-TUBB4A (1:1000, Abcam, Waltham, MA), rabbit anti-Vinculin (1:4000, Abcam, Waltham, MA), mouse anti-Actin (1:5000, Santa Cruz Biotechnology, Santa Cruz, CA), rabbit anti-GAPDH (1:5000, Cell Signaling, Danvers, MA), anti-mouse (1:20,000, ThermoFisher Scientific, Waltham, MA), anti-rabbit (1:20,000, ThermoFisher Scientific, Waltham, MA).

### F-actin morphology assay

Cells were seeded in 8-well chamber slides and incubated over night at 37 °C. Chambers were washed with 1 × PBS, fixed with 4% paraformaldehyde for 20 min at room temperature and permeabilized with 0.3% Triton-X (Biorad, Mississauga, Ontario). F-actin was stained using Rhodamine Phalloidin (1:400, ThermoFisher Scientific, Waltham, MA) for 30 min and counterstained with VECTASHIELD mounting media with DAPI (Vector Laboratories, Newark, CA). Images were taken with a Leica S DMi8 inverted fluorescence microscope. The major and minor axis of cells were measured using ImageJ.

### Immunofluorescence

Ex vivo tissues were incubated in Chen’s Media for 48–72 h at 37 °C prior to staining. Treatment with Tubacin (10 µM, MedChemExpress, Monmouth Junction, NJ), parthenolide (PTL, 20 µM, MedChemExpress, Monmouth Junction, NJ) or DMSO (Millipore Sigma, Oakville, Ontario) was added to ex vivo tissues in Chen’s Media for 4 h as positive controls for acetylated and detyrosinated ⍺-tubulin staining. Specimens and cell lines were fixed with 4% paraformaldehyde for 20 min and subsequently permeabilized with 0.3% Triton-X for 15 min. Samples were blocked with 3% Donkey Serum (Millipore Sigma, Oakville, Ontario), 3% Bovine Serum Albumin (Bioshop, Burlington, Ontario) and 0.3% Triton-X (Biorad, Mississauga, Ontario) for 1 h at room temperature. Primary antibodies were incubated over night at 4 °C. Secondary antibodies were incubated at room temperature for 1 h. Cells were counterstained with DAPI (300 nM, Millipore Sigma, Oakville, Ontario) for 10 min and mounted with ProLong Antifade mounting media (ThermoFisher Scientific, Waltham, MA). Specimens were gently unfolded using 1% hyaluronic acid onto a glass slide and counterstained with VECTASHEILD mounting media with DAPI (Vector Laboratories, Newark, CA). The primary and secondary antibodies and the concentrations used were: mouse anti-TUBB4A (1:100, Abcam, Waltham, MA), mouse anti-⍺-Tubulin (1:500, Millipore Sigma, Oakville, Ontario), mouse anti-acetylated ⍺-Tubulin (1:500, Millipore Sigma, Oakville, Ontario), rabbit anti-detyrosinated ⍺-Tubulin (1:100, Abcam, Waltham, MA), Cy3 anti-mouse (1:100, Jackson ImmunoResearch Labs, West Grove, PA), Alexa 647 anti-rabbit (1:500, ThermoFisher Scientific, Waltham, MA), Alexa 647 anti-mouse (1:500, ThermoFisher Scientific, Waltham, MA). Images were taken with a Leica S DMi8 inverted fluorescence microscope. Fluorescence intensity staining was quantified with ImageJ using the mean grey value over the total number of cells. A total of 5–8 images were quantified per sample.

### Statistical analysis

Statistical analysis was performed using a student *t*-test or either a one-way or two-way analysis of variance followed by a Dunnett’s post hoc test. A *p* value < 0.05 was considered statistically significant.

### Supplementary Information

Supplementary Figures.

Supplementary Table S1.

Supplementary Table S2.

Supplementary Table S3.

## Data Availability

The datasets generated during and/or analysed during the current study are available from the corresponding author on reasonable request.
