# Supplementary material for: Transcription factor 4 promotes increased corneal endothelial cellular migration by altering microtubules in Fuchs endothelial corneal dystrophy
Source: Sci Rep. 2024 May 4;14:10276. doi: 10.1038/s41598-024-61170-8 (PMC11069521; doi:10.1038/s41598-024-61170-8)
Supplement: Supplementary file 1 — Supplementary Figures. [file 41598_2024_61170_MOESM1_ESM.pdf]

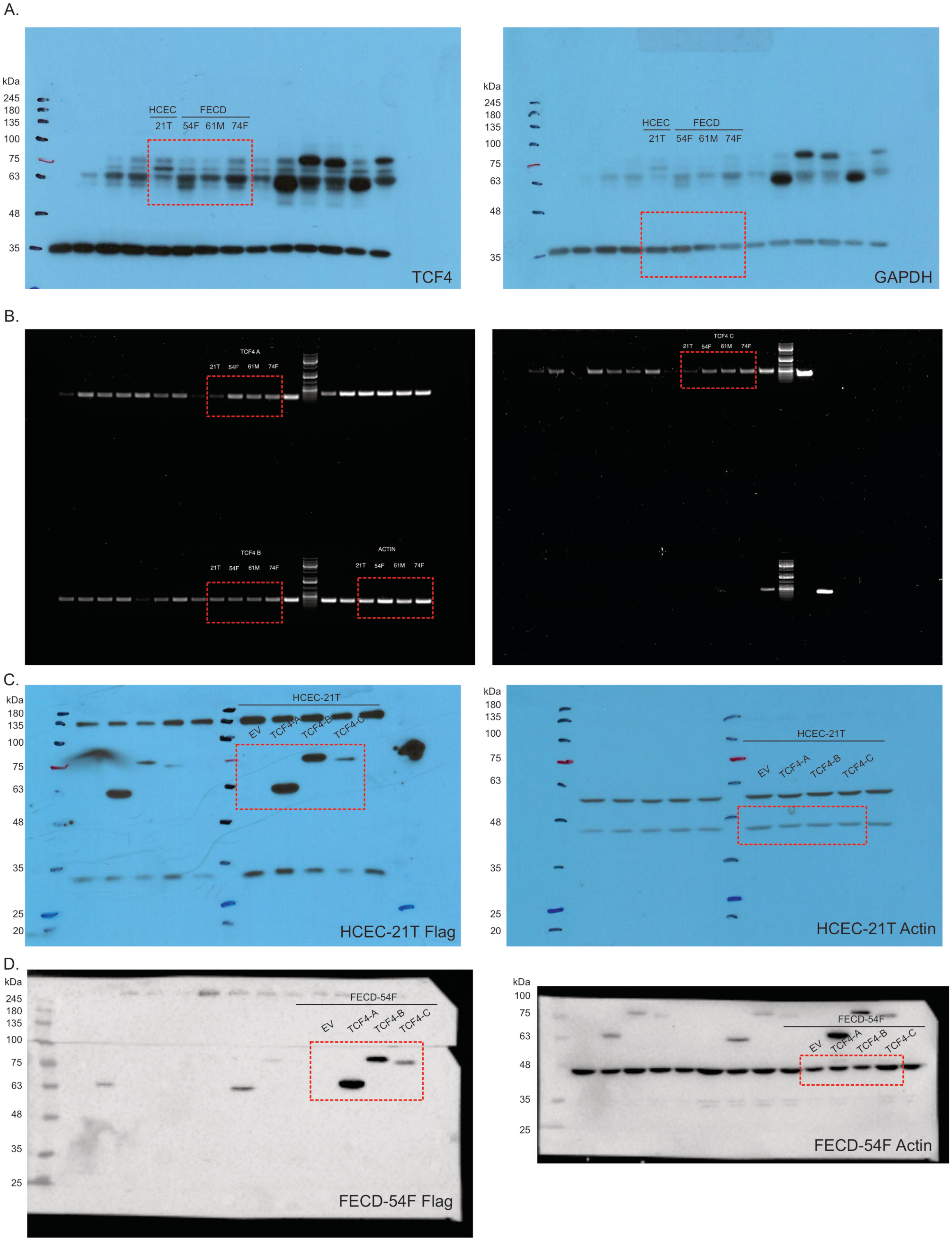

Supplementary Figure S1. Uncropped western blots and RT-PCR gels for Figure 1 for (A) TCF4 and GAPDH expression in CECs. (B) TCF4-A, -B, -C and actin mRNA expression in CECs. Flag and actin expression in HCEC-21T (C) and FECD-54F (D). Dashed red box marks section used in Figure 1.

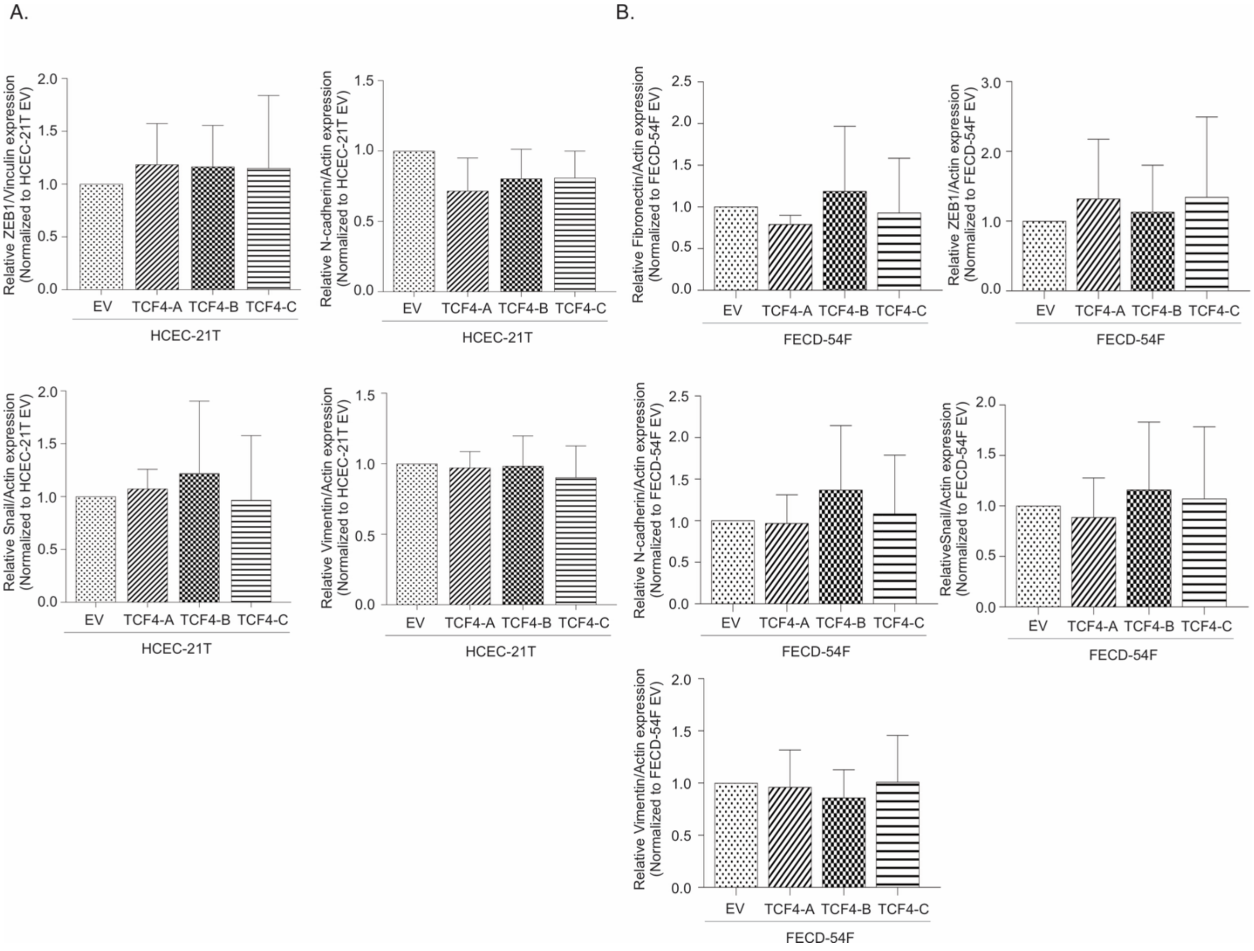

Supplementary Figure S2. Exogenous expression of TCF4 in CECs does not affect protein expression of common EMT markers. Densitometry quantification of protein expression (mean±SD) for common EMT markers in (A) HCEC-21T and (B) FECD-54F. β-actin or Vinculin was used as an internal control. SD = standard deviation.

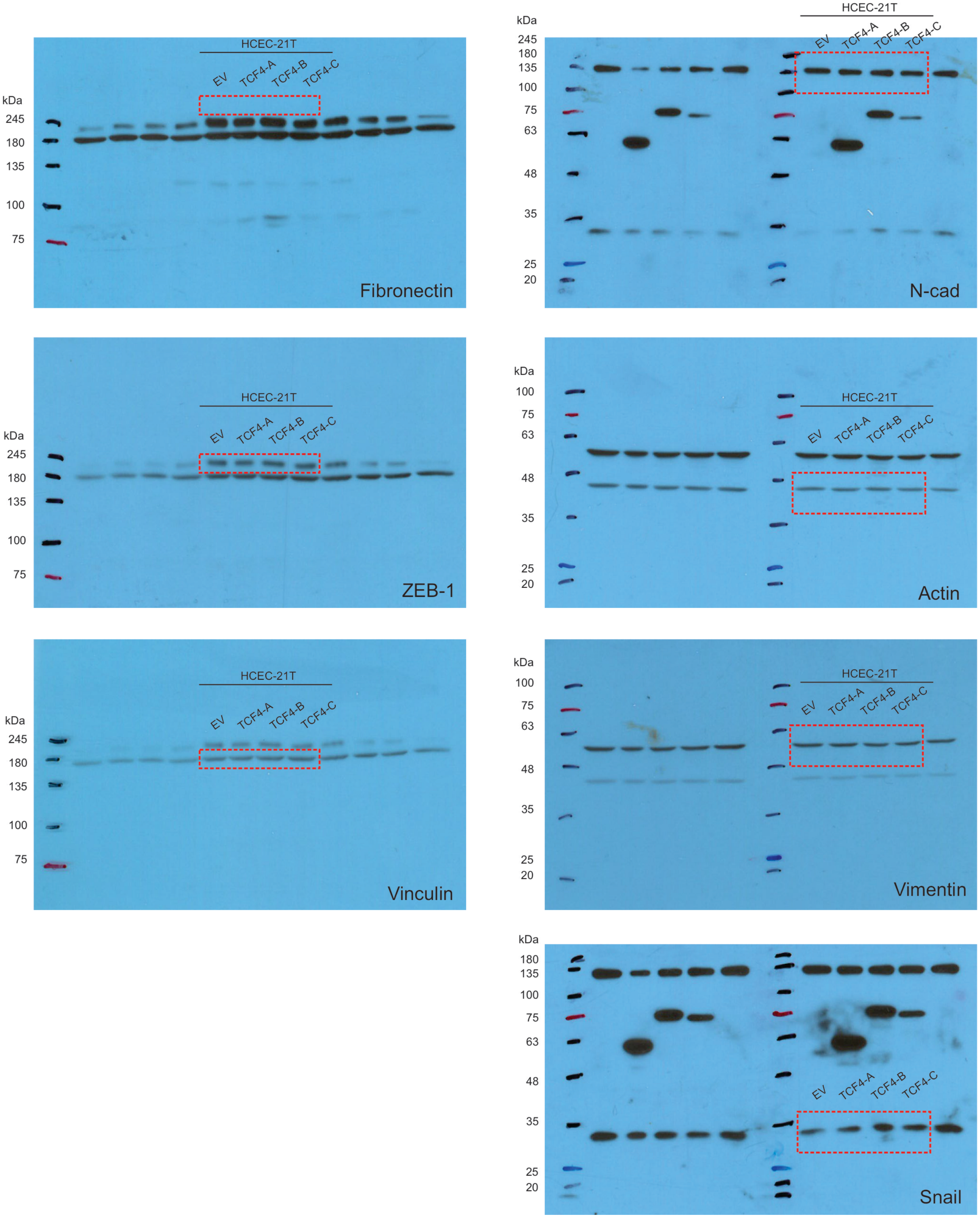

Supplementary Figure S3. Uncropped western blots for Figure 2 for EMT markers in HCEC-21T. Dashed red box marks section used in Figure 2.

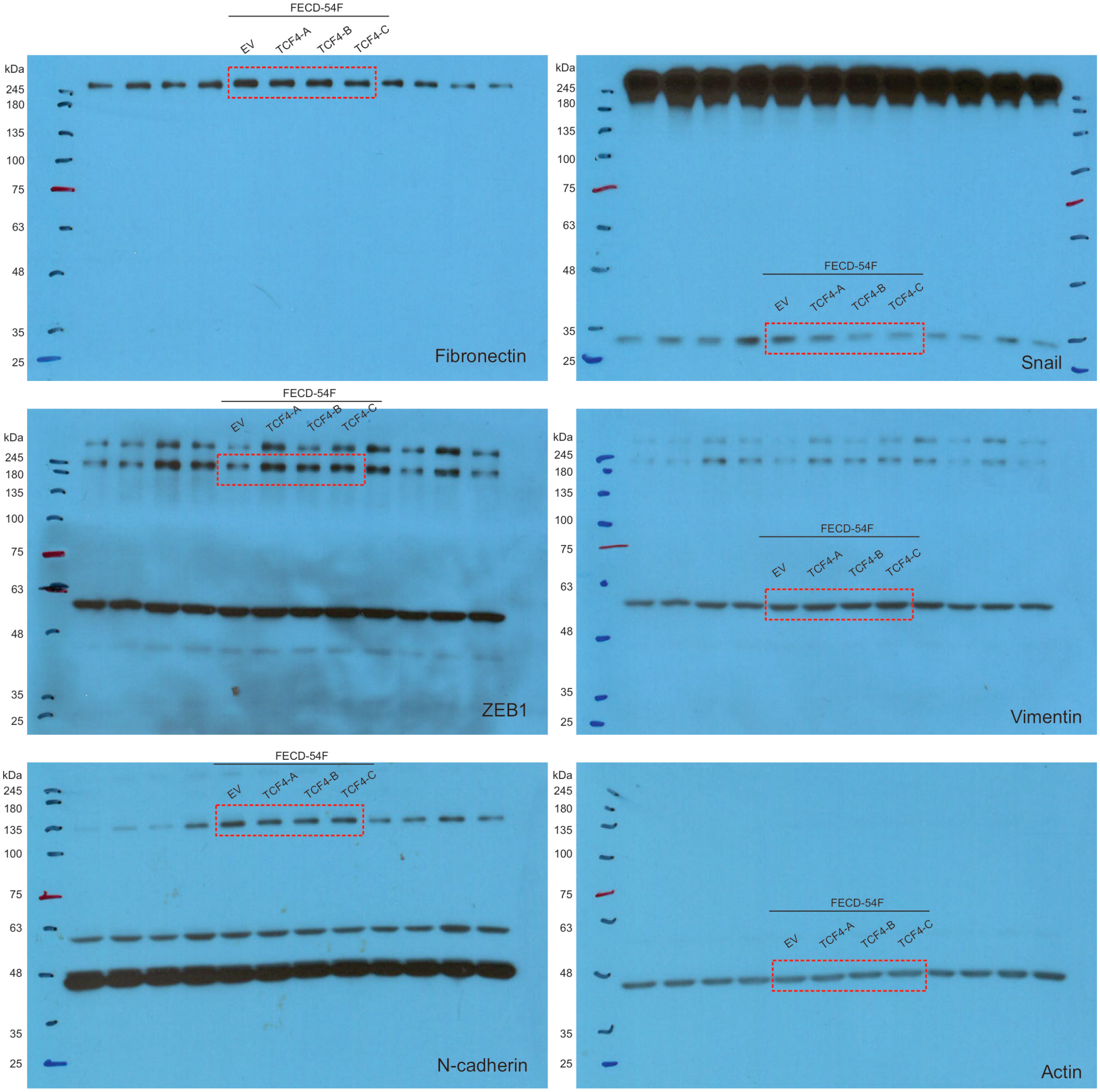

Supplementary Figure S4. Uncropped western blots for Figure 2 for EMT markers in FECD-54F. Dashed red box marks section used in Figure 2.

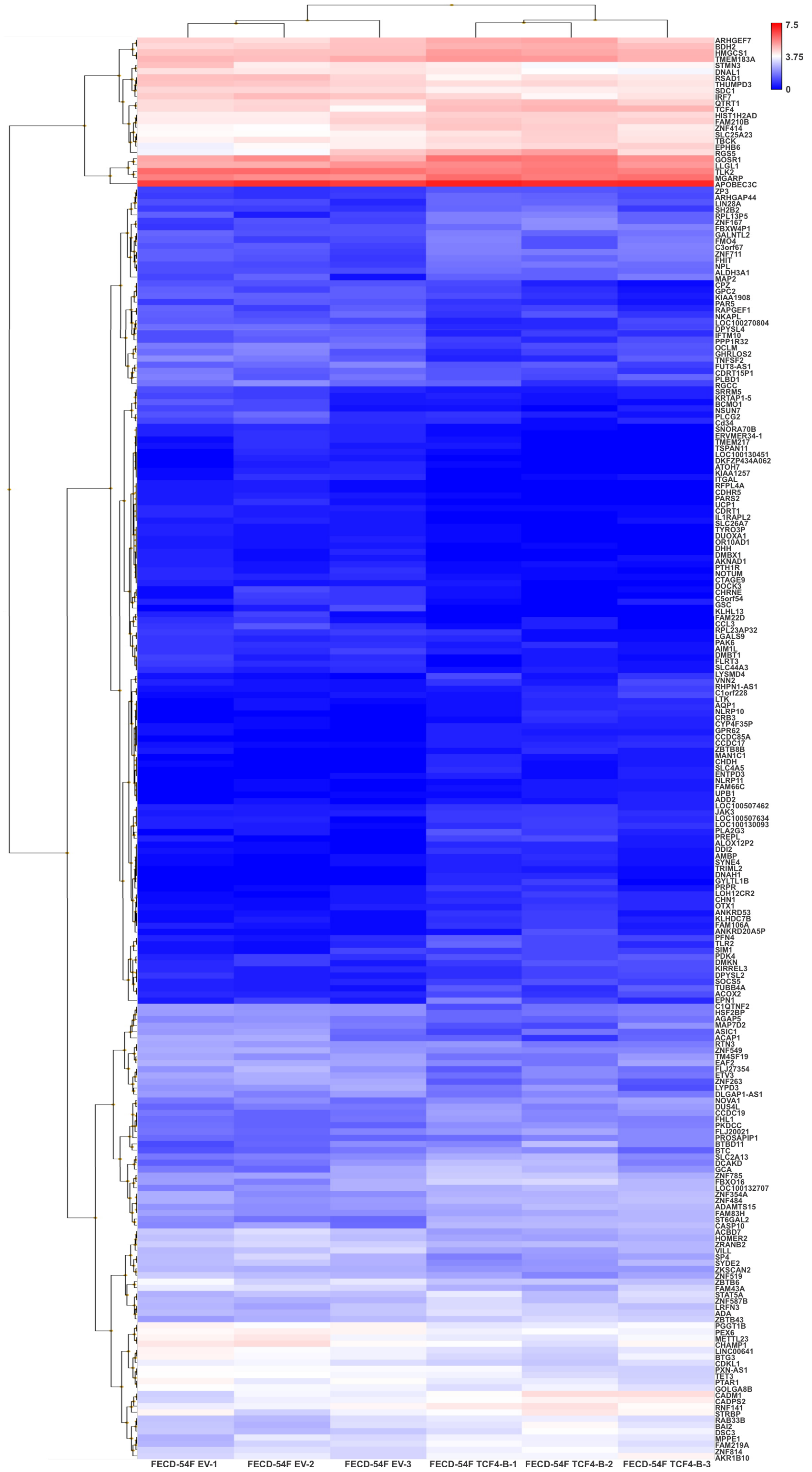

Supplementary Figure S5. Heatmap clustering of 227 differentially expressed gene changes in FECD-54F over expressing TCF4-B (n=3) compared to empty vector control (n=3) after stimulating cell migration with a scratch.

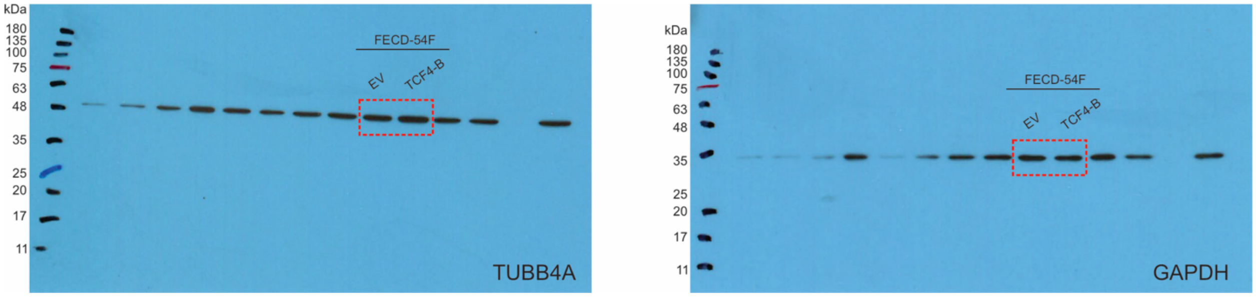

Supplementary Figure S6. Uncropped western blots for Figure 5 for TUBB4A and GAPDH expression. Dashed red box marks section used in Figure 5.

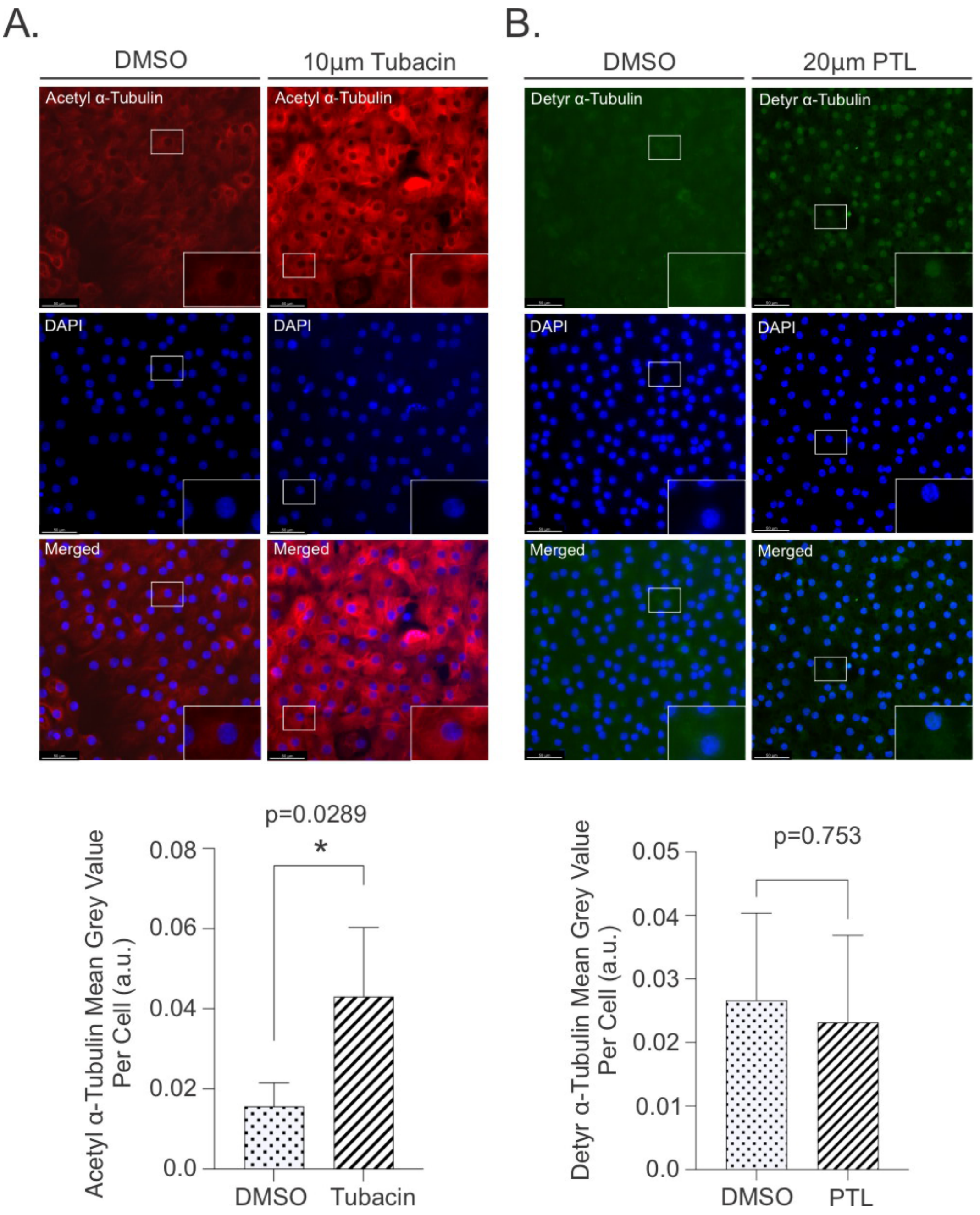

Supplementary Figure S7. Tubacin and PTL as positive controls to alter acetylated and detyrosinated α-tubulin expression in normal *ex vivo* specimens. (A) Representative images (top) with 2.5x magnification (inset) of acetylated α-tubulin expression after incubating with 10μM Tubacin for 4 hours compared to DMSO. Quantification (bottom) of fluorescence intensity (mean$\pm$SD) are shown for DMSO (n=4) and Tubacin (n=3). (B) Representative images (top) with 2.5x magnification (inset) of detyrosinated α-tubulin expression after incubating with 20μM PTL for 4 hours compared to DMSO. Quantification of fluorescence intensity (mean$\pm$SD) are shown for DMSO (n=4) and PTL (n=3). *:$p < 0.05$ by two tailed student *t*-test. SD = standard deviation. Scale bar = 50 μm.
