# Supplementary material for: Transcription factor 4 promotes increased corneal endothelial cellular migration by altering microtubules in Fuchs endothelial corneal dystrophy
Source: Sci Rep. 2024 May 4;14:10276. doi: 10.1038/s41598-024-61170-8 (PMC11069521; doi:10.1038/s41598-024-61170-8)
Supplement: Supplementary file 2 — Supplementary Table S1. [file 41598_2024_61170_MOESM2_ESM.pdf]

Supplementary Table S1. Gene expression changes for TCF4 isoform B over expression in FECD-54F

Gene Symbol	RefSeq	FC*	Gene Name
RGS5	NM_003617	2.23	regulator of G-protein signaling 5
PREPL	NM_001171603	2.09	prolyl endopeptidase-like
DCAKD	NM_001128631	2.01	dephospho-CoA kinase domain containing
TUBB4A	NM_006087	1.95	tubulin beta 4A class IVa
ST6GAL2	NM_032528	1.91	ST6 beta-galactosamide alpha-2 6-sialyltranferase 2
RPL13P5	NR_002803	1.88	ribosomal protein L13 pseudogene 5
PLA2G3	NM_015715	1.82	phospholipase A2 group III
FBXO16	NM_172366	1.8	F-box protein 16
ZNF167	NM_018651	1.79	zinc finger with KRAB and SCAN domains 7
CADM1	NM_014333	1.76	cell adhesion molecule 1
EPN1	NM_001130071	1.71	epsin 1
GCA	NM_012198	1.71	grancalcin EF-hand calcium binding protein
FMO4	NM_002022	1.69	flavin containing monooxygenase 4
TLR2	NM_003264	1.69	toll-like receptor 2
SIM1	NM_005068	1.66	single-minded family bHLH transcription factor 1
CASP10	NM_032977	1.64	caspase 10 apoptosis-related cysteine peptidase
C3orf67	NM_198463	1.62	chromosome 3 open reading frame 67
FBXW4P1	NR_033408	1.62	F-box and WD repeat domain containing 4 pseudogene 1
ZNF414	NM_001146175	1.59	zinc finger protein 414
GOSR1	NM_004871	1.56	golgi SNAP receptor complex member 1
ANKRD53	NM_001115116	1.54	ankyrin repeat domain 53
NPL	NM_030769	1.54	N-acetylneuraminate pyruvate lyase dihydrodipicolinate synthase
DPYSL2	NM_001197293	1.53	dihydropyrimidinase-like 2
DSC3	NM_001941	1.53	desmocollin 3
FHIT	NM_001166243	1.53	fragile histidine triad
PKDCC	NM_138370	1.52	protein kinase domain containing cytoplasmic
SLC2A13	NM_052885	1.52	solute carrier family 2 facilitated glucose transporter member 13
SH2B2	NM_020979	1.51	SH2B adaptor protein 2
TCF4	NM_001083962	1.51	transcription factor 4
DNAH1	NM_015512	1.5	dynein axonemal heavy chain 1

LYSMD4	NM_152449	1.5	LysM putative peptidoglycan-binding domain containing 4
ARHGEF7	NM_001113511	1.49	Rho guanine nucleotide exchange factor GEF 7
ZNF711	NM_021998	1.49	zinc finger protein 711
ZNF785	NM_152458	1.49	zinc finger protein 785
AQP1	NM_001185060	1.49	aquaporin 1 Colton blood group
ADAMTS15	NM_139055	1.48	ADAM metallopeptidase with thrombospondin type 1 motif 15
NLRP10	NM_176821	1.48	NLR family pyrin domain containing 10
BTC	NM_001729	1.47	betacellulin
PFN4	NM_199346	1.47	profilin family member 4
ZNF354A	NM_005649	1.47	zinc finger protein 354A
DUS4L	NM_181581	1.46	dihydrouridine synthase 4-like S. cerevisiae
ZNF587B	NM_001204818	1.46	zinc finger protein 587B
ACOX2	NM_003500	1.45	acyl-CoA oxidase 2 branched chain
ARHGAP44	NM_014859	1.45	Rho GTPase activating protein 44
CHN1	NM_001822	1.45	chimerin 1
GYLTL1B	NM_152312	1.45	glycosyltransferase-like 1B
C1orf228	NM_001145636	1.44	chromosome 1 open reading frame 228
CCDC19	NM_012337	1.44	coiled-coil domain containing 19
RAB33B	NM_031296	1.44	RAB33B member RAS oncogene family
DMKN	NM_033317	1.42	dermokine
EPHB6	NM_004445	1.42	EPH receptor B6
FLJ20021	NR_033874	1.42	uncharacterized LOC90024
LLGL1	NM_004140	1.42	lethal giant larvae homolog 1 Drosophila
ZBTB43	NM_001135776	1.42	zinc finger and BTB domain containing 43
AKR1B10	NM_020299	1.4	aldo-keto reductase family 1 member B10 aldose reductase
CCDC85A	NM_001080433	1.4	coiled-coil domain containing 85A
ENTPD3	NM_001248	1.4	ectonucleoside triphosphate diphosphohydrolase 3
MAP2	NM_002374	1.4	microtubule-associated protein 2
PDK4	NM_002612	1.4	pyruvate dehydrogenase kinase isozyme 4
GALNTL2	NM_054110	1.39	polypeptide N-acetylgalactosaminyltransferase 15
PRLR	NM_000949	1.39	prolactin receptor
VNN2	NM_004665	1.39	vanin 2
LOC100132707	NR_024476	1.39	PAXIP1 antisense RNA 2

RNF141	NM_016422	1.38	ring finger protein 141
STAT5A	NM_003152	1.38	signal transducer and activator of transcription 5A
CRB3	NM_174881	1.37	crumbs homolog 3 Drosophila
LTK	NM_002344	1.37	leukocyte receptor tyrosine kinase
SLC4A5	NM_021196	1.37	solute carrier family 4 sodium bicarbonate cotransporter member 5
CCDC17	NM_001114938	1.36	coiled-coil domain containing 17
FAM106A	NR_026809	1.36	family with sequence similarity 106 member A
NOVA1	NM_002515	1.36	neuro-oncological ventral antigen 1
PROSAPIP1	NM_014731	1.36	leucine zipper tumor suppressor family member 3
SOCS5	NM_014011	1.36	suppressor of cytokine signaling 5
ZP3	NM_001110354	1.36	zona pellucida glycoprotein 3 sperm receptor
ALOX12P2	NR_002710	1.36	arachidonate 12-lipoxygenase pseudogene 2
BDH2	NM_020139	1.35	3-hydroxybutyrate dehydrogenase type 2
BTBD11	NM_001018072	1.35	BTB POZ domain containing 11
HIST1H2AD	NM_021065	1.35	histone cluster 1 H2ad
JAK3	NM_000215	1.35	Janus kinase 3
LOH12CR2	NR_024061	1.35	loss of heterozygosity 12 chromosomal region 2 non-protein coding
TMEM183A	NM_138391	1.35	transmembrane protein 183A
MGARP	NM_032623	1.34	mitochondria-localized glutamic acid-rich protein
FAM219A	NM_001184940	1.34	family with sequence similarity 219 member A
FAM210B	NM_080821	1.34	family with sequence similarity 210 member B
FAM83H	NM_198488	1.34	family with sequence similarity 83 member H
MPPE1	NM_023075	1.34	metallophosphoesterase 1
BAI2	NM_001703	1.33	brain-specific angiogenesis inhibitor 2
DDI2	NM_032341	1.33	DNA-damage inducible 1 homolog 2 S. cerevisiae
ZBTB8B	NM_001145720	1.33	zinc finger and BTB domain containing 8B
ZNF814	NM_001144989	1.33	zinc finger protein 814
MAN1C1	NM_020379	1.32	mannosidase alpha class 1C member 1
ZNF484	NM_031486	1.32	zinc finger protein 484
ALDH3A1	NM_001135168	1.31	aldehyde dehydrogenase 3 family member A1
ADD2	NM_001185054	1.3	adducin 2 beta
CADPS2	NM_001009571	1.3	Ca-dependent secretion activator 2
CHDH	NM_018397	1.3	choline dehydrogenase

FAM66C	NR_026788	1.3	family with sequence similarity 66 member C
GPR62	NM_080865	1.3	G protein-coupled receptor 62
LRFN3	NM_024509	1.3	leucine rich repeat and fibronectin type III domain containing 3
NLRP11	NM_145007	1.3	NLR family pyrin domain containing 11
STRBP	NM_018387	1.3	spermatid perinuclear RNA binding protein
ANKRD20A5P	NR_040113	1.3	ankyrin repeat domain 20 family member A5 pseudogene
LOC100130093	NR_024485	1.3	synaptosome associated protein 47
LIN28A	NM_024674	1.29	lin-28 homolog A C. elegans
APOBEC3C	NM_014508	1.28	apolipoprotein B mRNA editing enzyme catalytic polypeptide-like 3C
FHL1	NM_001159702	1.28	four and a half LIM domains 1
KIRREL3	NM_032531	1.28	kin of IRRE like 3 Drosophila
LOC100507462	NM_001242740	1.28	CCDC28A antisense RNA 1
QTRT1	NM_031209	1.28	queuine tRNA-ribosyltransferase 1
TRIML2	NM_173553	1.28	tripartite motif family-like 2
UPB1	NM_016327	1.28	ureidopropionase beta
ADA	NM_000022	1.27	adenosine deaminase
AMBP	NM_001633	1.27	alpha-1-microglobulin bikunin precursor
RHPN1-AS1	NR_026785	1.27	RHPN1 antisense RNA 1 head to head
CYP4F35P	NR_026756	1.27	cytochrome P450 family 4 subfamily F polypeptide 35 pseudogene
HMGCS1	NM_002130	1.27	3-hydroxy-3-methylglutaryl-CoA synthase 1 soluble
LOC100507634	NR_038320	1.27	uncharacterized LOC100507634
SYNE4	NM_001039876	1.26	spectrin repeat containing nuclear envelope family member 4
SLC25A23	NM_024103	1.26	solute carrier family 25 mitochondrial carrier phosphate carrier member 23
TBCK	NM_001163435	1.26	TBC1 domain containing kinase
OTX1	NM_014562	1.25	orthodenticle homeobox 1
CDRT1	NM_006382	-1.26	CMT1A duplicated region transcript 1
DKFZP434A062	NR_026964	-1.26	uncharacterized LOC26102
PAK6	NM_001128628	-1.26	p21 protein Cdc42 Rac -activated kinase 6
THUMPD3	NM_015453	-1.26	THUMP domain containing 3
TSPAN11	NM_001080509	-1.26	tetraspanin 11
ZKSCAN2	NM_001012981	-1.26	zinc finger with KRAB and SCAN domains 2

ZRANB2	NM_203350	-1.26	zinc finger RAN-binding domain containing 2
AKNAD1	NM_152763	-1.27	AKNA domain containing 1
SDC1	NM_002997	-1.27	syndecan 1
ZNF519	NM_145287	-1.27	zinc finger protein 519
DHH	NM_021044	-1.28	desert hedgehog
HOMER2	NM_199330	-1.28	homer homolog 2 Drosophila
PTH1R	NM_001184744	-1.28	parathyroid hormone 1 receptor
TLK2	NM_006852	-1.28	tousled-like kinase 2
PEX6	NM_000287	-1.29	peroxisomal biogenesis factor 6
PTAR1	NM_001099666	-1.29	protein prenyltransferase alpha subunit repeat containing 1
STMN3	NM_015894	-1.29	stathmin-like 3
CD34	NM_001025109	-1.3	CD34 molecule
DNAL1	NM_031427	-1.3	dynein axonemal light chain 1
DOCK3	NM_004947	-1.3	dedicator of cytokinesis 3
DUOXA1	NM_144565	-1.3	dual oxidase maturation factor 1
GOLGA8B	NM_001023567	-1.3	golgin A8 family member B
OR10AD1	NM_001004134	-1.3	olfactory receptor family 10 subfamily AD member 1
PARS2	NM_152268	-1.3	prolyl-tRNA synthetase 2 mitochondrial putative
SLC26A7	NM_134266	-1.3	solute carrier family 26 anion exchanger member 7
TYRO3P	NR_028510	-1.3	TYRO3P protein tyrosine kinase pseudogene
ATOH7	NM_145178	-1.31	atonal homolog 7 Drosophila
CHAMP1	NM_001164144	-1.31	chromosome alignment maintaining phosphoprotein 1
CDHR5	NM_021924	-1.32	cadherin-related family member 5
LINC00641	NR_038970	-1.32	long intergenic non-protein coding RNA 641
PLBD1	NM_024829	-1.32	phospholipase B domain containing 1
RFPL4A	NM_001145014	-1.32	ret finger protein-like 4A
SLC44A3	NM_001114106	-1.32	solute carrier family 44 member 3
AIM1L	NM_001039775	-1.33	absent in melanoma 1-like
IL1RAPL2	NM_017416	-1.33	interleukin 1 receptor accessory protein-like 2
UCP1	NM_021833	-1.33	uncoupling protein 1 mitochondrial proton carrier
AGAP5	NM_001144000	-1.34	ArfGAP with GTPase domain ankyrin repeat and PH domain 5

METTL23	NM_001206984	-1.34	methyltransferase like 23
SNORA70B	NR_003707	-1.34	small nucleolar RNA H ACA box 70B
LOC100130451	NM_001242575	-1.35	SPAG16 divergent transcript
NOTUM	NM_178493	-1.35	notum pectinacetylesterase homolog Drosophila
CDKL1	NM_004196	-1.35	cyclin-dependent kinase-like 1 CDC2-related kinase
FUT8-AS1	NR_024334	-1.35	FUT8 antisense RNA 1
CDRT15P1	NR_003261	-1.36	CMT1A duplicated region transcript 15 pseudogene 1
VILL	NM_015873	-1.36	villin-like
CTAGE9	NM_001145659	-1.37	CTAGE family member 9
LYPD3	NM_014400	-1.37	LY6 PLAUR domain containing 3
PPP1R32	NM_145017	-1.37	protein phosphatase 1 regulatory subunit 32
BTG3	NM_006806	-1.39	BTG family member 3
IRF7	NM_001572	-1.39	interferon regulatory factor 7
KRTAP1-5	NM_031957	-1.39	keratin associated protein 1-5
RTN3	NM_201428	-1.39	reticulon 3
EAF2	NM_018456	-1.4	ELL associated factor 2
GSC	NM_173849	-1.4	goosecoid homeobox
PGGT1B	NM_005023	-1.4	protein geranylgeranyltransferase type I beta subunit
SP4	NM_003112	-1.4	Sp4 transcription factor
DLGAP1-AS1	NR_024101	-1.4	DLGAP1 antisense RNA 1
ACBD7	NM_001039844	-1.41	acyl-CoA binding domain containing 7
FLJ27354	NR_033981	-1.41	LRRC8C divergent transcript
FLRT3	NM_013281	-1.41	fibronectin leucine rich transmembrane protein 3
HSF2BP	NM_007031	-1.41	heat shock transcription factor 2 binding protein
DMBT1	NM_007329	-1.42	deleted in malignant brain tumors 1
SRRM5	NM_001145641	-1.42	serine arginine repetitive matrix 5
ZBTB6	NM_006626	-1.42	zinc finger and BTB domain containing 6
C1QTNF2	NM_031908	-1.43	C1q and tumor necrosis factor related protein 2
LGALS9	NM_009587	-1.43	lectin galactoside-binding soluble 9
DMBX1	NM_147192	-1.44	diencephalon mesencephalon homeobox 1
KLHL13	NM_001168299	-1.44	kelch-like family member 13
TET3	NM_144993	-1.44	tet methylcytosine dioxygenase 3
TMEM217	NM_145316	-1.44	transmembrane protein 217
KIAA1257	NM_020741	-1.46	cilia and flagella associated protein 92 (putative)

ITGAL	NM_002209	-1.47	integrin alpha L antigen CD11A p180 lymphocyte function-associated antigen 1 alpha polypeptide
ERVMER34-1	NM_001242690	-1.48	endogenous retrovirus group MER34 member 1
SYDE2	NM_032184	-1.48	synapse defective 1 Rho GTPase homolog 2 C. elegans
ZNF549	NM_001199295	-1.48	zinc finger protein 549
CCL3	NM_002983	-1.51	chemokine C-C motif ligand 3
RAPGEF1	NM_198679	-1.51	Rap guanine nucleotide exchange factor GEF 1
NKAPL	NM_001007531	-1.53	NFKB activating protein-like
PXN-AS1	NR_038924	-1.54	PXN antisense RNA 1
ZAR1L	NM_001136571	-1.54	zygote arrest 1-like
FAM43A	NM_153690	-1.56	family with sequence similarity 43 member A
GHRLOS2	NR_026829	-1.56	long intergenic non-protein coding RNA 852
RSAD1	NM_018346	-1.56	radical S-adenosyl methionine domain containing 1
C5orf54	NM_022090	-1.57	chromosome 5 open reading frame 54
KIAA1908	NR_021487	-1.57	PSMG3 antisense RNA 1 (head to head)
TM4SF19	NM_001204897	-1.59	transmembrane 4 L six family member 19
CPZ	NM_001014447	-1.6	carboxypeptidase Z
PLCG2	NM_002661	-1.6	phospholipase C gamma 2 phosphatidylinositol-specific
FAM22D	NM_001009610	-1.61	NUT family member 2D
ETV3	NM_001145312	-1.62	ets variant 3
RGCC	NM_014059	-1.63	regulator of cell cycle
OCLM	NM_022375	-1.63	oculomedin
GPC2	NM_152742	-1.67	glypican 2
LOC100270804	NR_026885	-1.67	lysosome cell death regulator
RPL23AP32	NR_002229	-1.67	ribosomal protein L23a pseudogene 32
IFITM10	NM_001170820	-1.69	interferon induced transmembrane protein 10
PAR5	NR_022008	-1.69	14-3-3 domain-containing protein;14-3-3-like protein 1
BCMO1	NM_017429	-1.7	beta-carotene 15 15p-monooxygenase 1
NSUN7	NM_024677	-1.73	NOP2 Sun domain family member 7
CHRNE	NM_000080	-1.82	cholinergic receptor nicotinic epsilon muscle
ASIC1	NM_020039	-1.85	acid-sensing proton-gated ion channel 1
DPYSL4	NM_006426	-2.05	dihydropyrimidinase-like 4

ACAP1	NM_014716	-2.08	ArfGAP with coiled-coil ankyrin repeat and PH domains 1
MAP7D2	NM_001168465	-2.19	MAP7 domain containing 2
TNFSF4	NM_003326	-2.31	tumor necrosis factor ligand superfamily member 4
ZNF263	NM_005741	-2.31	zinc finger protein 263

* Fold Change relative to FECD-54F EV
