# Supplementary material for: Transcription factor 4 promotes increased corneal endothelial cellular migration by altering microtubules in Fuchs endothelial corneal dystrophy
Source: Sci Rep. 2024 May 4;14:10276. doi: 10.1038/s41598-024-61170-8 (PMC11069521; doi:10.1038/s41598-024-61170-8)
Supplement: Supplementary file 3 — Supplementary Table S2. [file 41598_2024_61170_MOESM3_ESM.pdf]

Supplementary Table S2. Patient and Donor Specimen Characteristics

Specimen	Age (yrs)	Sex	Endothelial Cell Density (cells/mm^2)	Cause of Death	Death to Preservation Time (hrs:mins)	IF Staining
Normal	62	F	2957	Stroke	10:30	TUBB4A
Normal	72	F	2494	Stroke	13:30	TUBB4A
Normal	30	M	N/A	Stroke	22:57	TUBB4A
Normal	64	F	2720	Congestive Heart Failure	7:30	α-Tubulin/F-actin
Normal	53	M	N/A	Aspiration Pneumonia	21:46	α-Tubulin/F-actin
Normal	65	F	N/A	Cancer	17:10	α-Tubulin/F-actin
Normal	73	F	3003	Stroke	18:25	Acetyl/Detyr α-Tubulin
Normal	57	M	2688	Cirrhosis	22:02	Acetyl/Detyr α-Tubulin
Normal	59	M	3003	Cancer	26:19	Acetyl/Detyr α-Tubulin
Normal	60	M	2646	Cancer	10:10	Acetyl/Detyr α-Tubulin
Normal	77	F	2262	Cancer	21:45	Acetyl/Detyr α-Tubulin
Normal	65	F	2079	Cancer	5:39	Acetyl/Detyr α-Tubulin
Normal	57	M	2392	Stroke	7:28	Acetyl/Detyr α-Tubulin
Normal	63	M	2584	Cancer	21:25	Acetyl/Detyr α-Tubulin
Normal	70	M	2548	Cirrhosis	24:35	Acetyl/Detyr α-Tubulin
FECD	83	M	N/A	N/A	N/A	TUBB4A
FECD	57	F	N/A	N/A	N/A	TUBB4A
FECD	81	M	N/A	N/A	N/A	TUBB4A
FECD	74	F	N/A	N/A	N/A	α-Tubulin/F-actin
FECD	72	F	N/A	N/A	N/A	α-Tubulin/F-actin
FECD	81	F	N/A	N/A	N/A	α-Tubulin/F-actin
FECD	75	F	N/A	N/A	N/A	Acetyl/Detyr α-Tubulin
FECD	75	M	N/A	N/A	N/A	Acetyl/Detyr α-Tubulin
FECD	67	F	N/A	N/A	N/A	Acetyl/Detyr α-Tubulin
