# Supplementary material for: Transcription factor 4 promotes increased corneal endothelial cellular migration by altering microtubules in Fuchs endothelial corneal dystrophy
Source: Sci Rep. 2024 May 4;14:10276. doi: 10.1038/s41598-024-61170-8 (PMC11069521; doi:10.1038/s41598-024-61170-8)
Supplement: Supplementary file 4 — Supplementary Table S3. [file 41598_2024_61170_MOESM4_ESM.pdf]

Supplementary Table S3. RT-PCR Primer

**Primers**	**Sequence 5'-3'**	**Annealing Temperature**
TCF4 A	CATACACAATCCCGGGCATG<br>CCCAACATTCCTGCATAGCC	58°C
TCF4 B	TAGGGACGGACAAAGAGCTG<br>TGCCCATATCCATGTCACCT	58°C
TCF4 C	CTGGAGCAGGACTGAGTACC<br>TGCCCATATCCATGTCACCT	58°C
Actin	ATTGCCGACAGGATGCAGAAG<br>CTGTCACCTTCACCGTTCCAG	58°C
